# Mapping Transmission Dynamics and Drug Resistance Surveillance in the Cyprus HIV-1 Epidemic (2017–2021)

**DOI:** 10.3390/v16091449

**Published:** 2024-09-11

**Authors:** Cicek Topcu, Bram Vrancken, Johana Hezka Rodosthenous, David van de Vijver, Georgios Siakallis, Philippe Lemey, Leondios G. Kostrikis

**Affiliations:** 1 Laboratory of Biotechnology and Molecular Virology, Department of Biological Sciences, University of Cyprus, 2109 Nicosia, Cyprus; 2 Spatial Epidemiology Lab (SpELL), Université Libre de Bruxelles, 1050 Bruxelles, Belgium; 3 Laboratory of Clinical and Epidemiological Virology, Department of Microbiology, Immunology and Transplantation, Rega Institute, KU Leuven, 3000 Leuven, Belgium; 4 Department of Viroscience, Erasmus University Medical Centre, 3015 GD Rotterdam, The Netherlands; 5 AIDS Clinic, Larnaca General Hospital, 6043 Larnaca, Cyprus; 6 Cyprus Academy of Sciences, Letters, and Arts, 1011 Nicosia, Cyprus

**Keywords:** human immunodeficiency virus type 1 (HIV-1), HIV-1 molecular epidemiology, HIV-1 transmitted drug resistance, HIV-1 phylogenetics, HIV-1 phylodynamics, HIV-1 phylogeographics, Cyprus

## Abstract

The human immunodeficiency virus type 1 (HIV-1) epidemic has been a major public health threat on a global scale since the early 1980s. Despite the introduction of combination antiretroviral therapy (cART), the incidence of new HIV-1 infections continues to rise in some regions around the world. Thus, with the continuous transmission of HIV-1 and the lack of a cure, it is imperative for molecular epidemiological studies to be performed, to monitor the infection and ultimately be able to control the spread of this virus. This work provides a comprehensive molecular epidemiological analysis of the HIV-1 infection in Cyprus, through examining 305 HIV-1 sequences collected between 9 March 2017 and 14 October 2021. Employing advanced statistical and bioinformatic techniques, the research delved deeply into understanding the transmission dynamics of the HIV-1 epidemic in Cyprus, as well as the monitoring of HIV-1’s genetic diversity and the surveillance of transmitted drug resistance. The characterization of Cyprus’s HIV-1 epidemic revealed a diverse landscape, comprising 21 HIV-1 group M pure subtypes and circulating recombinant forms (CRFs), alongside numerous uncharacterized recombinant strains. Subtypes A1 and B emerged as the most prevalent strains, followed by CRF02_AG. The findings of this study also revealed high levels of transmitted drug resistance (TDR) patterns, raising concerns for the efficacy of cART. The demographic profiles of individuals involved in HIV-1 transmission underscored the disproportionate burden borne by young to middle-aged Cypriot males, particularly those in the MSM community, who reported contracting the virus in Cyprus. An assessment of the spatiotemporal evolutionary dynamics illustrated the global interconnectedness of HIV-1 transmission networks, implicating five continents in the dissemination of strains within Cyprus: Europe, Africa, Asia, North America, and Oceania. Overall, this study advances the comprehension of the HIV-1 epidemic in Cyprus and highlights the importance of understanding HIV-1’s transmission dynamics through continuous surveillance efforts. Furthermore, this work emphasizes the critical role of state-of-the-art bioinformatics analyses in addressing the challenges posed by HIV-1 transmission globally, laying the groundwork for public health interventions aimed at curbing its spread and improving patient outcomes.

## 1. Introduction

The human immunodeficiency virus type 1 (HIV-1) epidemic struck the world over four decades ago, and, since then, it has been an unceasing major global health threat. According to the World Health Organization (WHO), by the end of 2021, approximately 84.2 million people had been diagnosed with an HIV infection and 40.1 million people had died due to acquired immunodeficiency syndrome (AIDS)-related diseases since the beginning of the pandemic in 1981 [1,2]. As reported by UNAIDS, in 2021, there were 38.4 million people living with the infection, about 1.5 million people were newly affected by HIV, and 650,000 had people died due to AIDS-related diseases [3].

Despite the development and remarkable success of combination antiretroviral therapy (cART), which has led to a considerable decrease in AIDS-related mortality and a longer life span through achieving an almost undetectable viral load, HIV-1 transmission continues to be a serious worldwide public health issue, with even higher transmission rates in some regions around the world [4,5]. Among these regions is the WHO Eastern Mediterranean region (EMR), where a 47% increase was observed in the incidence of new HIV infections in 2019 compared to 2010, along with a 57% increase in AIDS-related mortality [6,7,8,9]. Similarly, a continuous rise in new HIV infections has taken its toll on Cyprus. As such, an approximately 28% increase in the incidence of new HIV-1 infections in 2019 (*n* = 100) relative to 2018 (*n* = 78), a 6% increase in 2020 (*n* = 106) relative to 2019, and, most recently, a 40% increase in 2021 (*n* = 148) relative to 2020 have been recorded [10]. 

By 2019, in Cyprus, about 90% of all people diagnosed with an HIV-1 infection were prescribed cART. Regardless of the high percentage of HIV-1-affected individuals receiving therapy with high virologic suppression being achieved, the incidence of new HIV-1 infections in Cyprus is not decreasing. Moreover, the continuous global transmission of HIV-1, coupled with the fast viral turnover and high mutation frequency, has resulted in an elevated rate of molecular evolution and hence an extensive degree of viral genetic diversity, hindering the effective treatment and prevention of HIV-1 infection. 

HIV-1 has been classified into four groups (M, N, O, and P) and further subdivided into 10 distinct phylogenetic subtypes (A, B, C, D, F, G, H, J, K, and L) within the major group, M, each with varying prevalence around the globe [11,12,13]. In regions with diverse HIV-1 epidemics, coinfection with multiple distinct strains increases, facilitating the emergence of new recombinant strains and the formation of circulating recombinant forms (CRFs) through recombination processes [14]. At present, there are 157 well-documented CRFs in the Los Alamos HIV Sequence Database, including six previously identified in Cyprus: CRF04_cpx, CRF91_cpx, CRF129_56G, CRF130_A1B, CRF131_A1B, and CRF138_cpx [15,16,17,18,19]. Some of these subtypes have been confirmed to be more transmissible than others, allowing them to evade the host immune system, or more virulent, resulting in a more rapid rate of disease progression [20,21,22]. Additionally, the accumulation of HIV-1 drug resistance mutations allows the virus to escape antiretroviral treatment, jeopardizing all of the progress achieved so far in fighting the HIV-1 epidemic [23]. 

Throughout numerous years of comprehensive prospective studies, our research efforts have delved deeply into the molecular epidemiology of HIV-1 in Cyprus [18,19,24,25,26,27,28,29,30]. Our latest molecular epidemiology study, conducted between 2010 and 2012, shed light on a diverse array of HIV-1 strains circulating in Cyprus [27]. Notably, subtypes B (41.0%) and A1 (19.0%) were the predominant strains, followed by subtypes C (7.0%), F1 (8.0%), CRF02_AG (4%), and A2 (2.0%), other CRFs (7.0%), and uncharacterized HIV-1 recombinant forms (12%) [15]. These findings reflect the polyphyletic nature of the HIV-1 epidemic on the island, attributable to its strategic location, facilitating the introduction of various HIV-1 group M subtypes and CRFs. The study also unveiled the demographic composition of those affected, with 78% of the cohort being male. Moreover, the investigation into the transmission routes uncovered that the majority of infections, at 51%, were attributed to men who have sex with men (MSM) or homo-/bisexual contact (HBC), with heterosexual contact (HC) accounting for 33%. Concurrently, the three most recent investigations into transmitted drug resistance mutations in Cyprus have elucidated notably low prevalence, exhibiting one of the lowest levels of transmitted drug resistance on a global scale [24,26,27]. In essence, our research studies focusing on the molecular epidemiology not only underscore the evolving landscape of the HIV-1 epidemic in Cyprus but also provide critical data that are essential in guiding intervention strategies and public health initiatives at both local and global levels.

Therefore, it is of vital importance for prospective molecular epidemiology studies to estimate the spatiotemporal dynamics of the HIV-1 epidemic and illuminate the mutational background of HIV-1 among different countries/regions. Molecular epidemiology studies will not only improve upon our understanding of HIV, but they will also allow legislators to implement informed policies to safeguard public health. Through the implementation of appropriate policies, the findings of this prospective molecular epidemiology study will have a direct impact on the incidence of new HIV-1 infections, to achieve the goal of diminishing new HIV-1 infections and AIDS in Cyprus, through the identification and protection of the cohorts living under a substantial risk of HIV-1 infection.

## 2. Materials and Methods

### 2.1. Participant Enrollment, Data Collection, and Ethical Compliance

This study involved the investigation of 313 HIV-1 nucleotide sequences obtained from 313 individuals residing in Cyprus, all of whom were diagnosed with an HIV-1 infection. The data collection spanned from 9 March 2017 to 14 October 2021, as part of a prospective molecular epidemiology investigation. Ethical clearance for all experimental procedures conducted as part of this study was obtained from the Cyprus National Bioethics Committee (CNBC), with clearance granted for two distinct timeframes: from 9 March 2017 to 13 October 2019 (approval number EEBK EΠ 2017.01.23, approval date 20 February 2017), and from 14 October 2019 to 14 October 2021 (approval number EEBK EΠ 2019 71, approval date 2 September 2019).

To participate in the study, individuals were required to meet specific inclusion criteria. These criteria dictated that participants had to be consenting individuals who were either newly diagnosed with HIV-1 or chronically affected but, in both cases, they had not yet undergone antiretroviral therapy (reportedly antiretroviral-naïve). The chronic infection status was determined by the clinician, with participants considered chronic if they had been diagnosed with an HIV infection for more than six months. Additionally, participants were required to have an HIV-1 viral load equal to or exceeding 10^3^ RNA copies/mL plasma.

In order to safeguard the anonymity of the study participants, strict adherence to the guidelines and regulations established by both the CNBC and the Office of the Commissioner for Personal Data Protection in Cyprus was maintained. As a component of this procedure, all participants provided written informed consent. A structured questionnaire, administered by qualified medical personnel, was utilized to gather comprehensive clinical, epidemiological, behavioral, and demographic data from the participants. Additionally, to uphold the confidentiality of the participating individuals, a double-coding method was employed. The study participants and their corresponding blood samples were assigned unique hospital and laboratory identification numbers, respectively, with no traceability regarding the patient’s identity. 

At the Grigorios HIV Clinic of Larnaca General Hospital, the blood samples, consent forms, and questionnaires of the participating individuals were gathered and sent to the Laboratory of Biotechnology and Molecular Virology at the University of Cyprus each sample coded with a unique hospital identification number. Serving as the primary national clinical facility in Cyprus for the comprehensive care of people with HIV-1 (PWH), the Grigorios HIV Clinic provides a distinctive and invaluable opportunity to create a precise cohort reflecting the country’s HIV-1 epidemic. Each year, blood samples from approximately 70% of newly diagnosed individuals linked to care at the Grigorios HIV Clinic are sent to our laboratory, with an estimated consent rate of 95%. These high participation rates affirm the clinic’s pivotal role in shaping precise insights into the landscape of the HIV-1 epidemic in Cyprus.

### 2.2. Blood Sample Collection, Plasma Isolation, and HIV-1 RNA Extraction Protocol

Blood samples procured from participants were promptly transferred to the Laboratory of Biotechnology and Molecular Virology at the University of Cyprus for subsequent processing. Within two hours post-collection, plasma and peripheral blood mononuclear cells (PBMCs) were isolated utilizing established methodologies [29]. Subsequently, HIV-1 RNA extraction from plasma was carried out following a detailed protocol, as outlined in the previous literature [30].

### 2.3. RT-PCR Amplification and Sequencing

Each of the 313 HIV-1 RNA samples derived from 313 respective individuals underwent RT-PCR amplification and sequencing targeting the HIV-1 *pol* region (2253–5250 in the HXB2 genome), which encompasses the *protease (PR)*, *reverse transcriptase (RT)*, *integrase (IN)*, and partial *Vif* domains. The process was executed utilizing a validated touchdown HIV-1 *pol* RT-PCR assay [18,19,29,30]. As such, 305 of the 313 HIV-1 RNA extracts were successfully amplified, while the remaining eight samples were PCR-negative. The resultant PCR product, spanning 3259 base pairs, underwent purification and Sanger sequencing at Macrogen Europe (Amsterdam, the Netherlands) (https://dna.macrogen-europe.com/eng/, accessed on 5 March 2024). The RT-PCR assay was developed using HIV-1-specific primers, optimized for effective PCR amplification and sequencing. This tailored approach ensured robust coverage across various HIV-1 group M subtypes, CRFs, and recombinant strains. After receiving the sequencing primer amplicons from Macrogen Europe, validation was conducted, and the final consensus sequence was generated using the Geneious^®^ 11.1.4 software (https://www.geneious.com, accessed on 15 January 2024) in accordance with the prior published criteria [29]. 

### 2.4. HIV-1 Genotypic Subtyping and Drug Resistance Analysis

The subsequent determination of the HIV-1 genotypic subtypes employed the REGA HIV-1 subtyping tool, version 3.0 (REGA 3.0) [31]. The detection of drug resistance mutations was accomplished through the renowned HIVdb Program hosted by the Stanford University HIV Drug Resistance Database [32]. To ensure the thorough verification of the surveillance resistance mutations (SRMs) in our study, we consulted multiple sources [32,33,34]. This approach allowed us to accurately identify and report all relevant SRMs. In our study, resistance to HIV-1 antiretroviral drugs was rigorously assessed across four key antiretroviral drug classes: NRTIs, NNRTIs, PIs, and INSTIs. Within each class, we examined five, five, three, and five drugs, respectively. Specifically, for NRTIs, we examined Abacavir (ABC), Zidovudine (AZT), Emtricitabine (FTC), Lamivudine (3TC), and Tenofovir (TDF); for NNRTIs, we examined Efavirenz (EFV), Doravirine (DOR), Etravirine (ETR), Nevirapine (NVP), and Rilpivirine (RPV); for PIs, we examined Atazanavir/r (ATV/r), Darunavir/r (DRV/r), and Lopinavir/r (LPV/r); and for INSTIs, we examined Bictegravir (BIC), Dolutegravir (DTG), Elvitegravir (EVG), Raltegravir (RAL), and Cabotegravir (CAB). Resistance was categorized into four levels: potential low-level, low-level, intermediate, and high-level resistance.

### 2.5. Exploring Phylogenetic Patterns of HIV-1 pol Region Sequences

Following the initial sequence retrieval, an extensive phylogenetic investigation was undertaken on the 305 HIV-1 *pol* region sequences, employing well-established bioinformatics tools and methodologies [18]. This phylogenetic investigation was conducted against a comprehensive reference data set encompassing all known HIV-1 group M subtypes (A, B, C, D, F, G, H, J, K, and L) and circulating recombinant forms (CRFs) (RIP Alignment 2020) sourced from the Los Alamos HIV Sequence Database (http://www.hiv.lanl.gov (accessed on 12 February 2024)). Additionally, the reference data set was augmented through Basic Local Alignment Search Tool (BLAST) analyses using the HIV BLAST tool available through the Los Alamos HIV Sequence Database (www.hiv.lanl.gov/content/sequence/BASIC_BLAST/basic_blast.html (accessed on 12 February 2024)). Data set enrichment involved selecting the first top BLAST hit with the highest percentage similarity for each of the HIV-1 molecular clusters identified in the preliminary analyses, ensuring comprehensive coverage.

Initially, multiple sequence alignment (MSA) was constructed utilizing the ClustalW algorithm in the Molecular Evolutionary Genetics Analysis (MEGA X) software [35,36]. MSA was then employed to infer a maximum likelihood (ML) phylogenetic tree using the general time-reversible (GTR) substitution model with gamma-distributed rate heterogeneity in the MEGA X software [36,37,38]. Bootstrap resampling with 1000 replicates was performed to assess the robustness of the tree topology. Following tree generation, clustering analyses were executed using the Cluster Picker software (version 1.2.3), utilizing previously defined parameters as per published methodologies, with a genetic distance threshold of 0.045 and a bootstrap support threshold of 70% to assess the clustering [28,39]. Finally, the resulting phylogenetic tree was visualized and refined using the FigTree v1.4.3 (http://tree.bio.ed.ac.uk/software/figtree/) software (accessed on 12 February 2024), enabling the elucidation and interpretation of the evolutionary relationships among the analyzed sequences [40]. Notably, the identified phylogenetic clusters were classified as putative transmission clusters, adhering to stringent criteria that necessitated a minimum of three samples to cluster together. This classification approach followed established methodologies, ensuring robustness and reliability in identifying potential transmission events within the analyzed data set [28].

### 2.6. Statistical Analyses

To explore the factors contributing to clustering, a rigorous statistical approach was employed for the 305 HIV-1 samples that were successfully amplified and sequenced. Continuous variables underwent analysis through *t*-tests, while categorical variables were assessed using either a chi-square test or Fisher’s exact test, depending on their appropriateness. This methodology allowed for a comprehensive investigation into the determinants underlying the clustering phenomena. Statistical analyses were conducted using the R software (version 4.3.2) and the significance level was set at 5% [41].

### 2.7. Bioinformatics Analysis

#### 2.7.1. Data Set Compilation

To delineate clusters that pertained to HIV-1 transmission within Cyprus, the newly generated sequences were complemented with a selection of publicly available data. To this end, all HIV-1 sequences from NCBI GenBank available on 28 September 2021 were used to construct a local BLAST database [42,43]. The latter was queried with the newly obtained cohort sequences that were subtyped as A1, A2, B, C, F1, G, and CRF02_AG by REGA 3.0 with the settings max_target_seqs 25, e-value 1, and word size 10 [31]. Different alignment scores are optimal for the detection of homologues at different evolutionary distances [44]. To obtain comprehensive background data sets, the similarity search was repeated with all combinations of match/mismatch scores in BLASTN that are optimal for the detection of sequences with 90% or more similarity. After the deduplication of the hits, lab strains were removed and only the earliest sampled sequence from longitudinal sets was kept. Similarly, a single sequence was kept from sets of clonal sequences sampled at the same time point. Subtype- and CRF-specific data sets were aligned with MAFFT v7.475, and the genome region covering HXB2 positions 2253–5250 was selected and manually refined using AliView [45,46]. For CRF91_cpx, CRF129_56G, CRF130_A1B, CRF131_A1B, and CRF138_cpx, separate data sets were created for the non-recombinant subregions (Table 1), following the above-detailed approach. An overview of the data set sizes is presented in Table 2. None of the aligned and edited data sets possessed significant recombination signals, as evaluated with the Phi-test (*p* > 0.05) [47]. Location and sampling date information was extracted from the GenBank records, and, if missing, we attempted to retrieve this from the Los Alamos HIV database or the publication(s) linked to the sequences.

#### 2.7.2. Timed Phylogeographic Inference

Time-stamped phylogenetic trees were estimated using the Bayesian Evolutionary Analysis by Sampling Trees (BEAST) software v1.10 [48]. For all data sets, a GTR model with Γ distributed among site rate variation was used to describe the substitution process and a flexible non-parametric model was fitted to model demographic changes [38,49]. The rate of evolution was estimated using a relaxed clock model with rates drawn from an underlying lognormal distribution [50]. For the largest data sets, A1, B, and C (see Table 2), we capitalized on previously published data with strong temporal signals to calibrate the molecular clock. Specifically, a normal distribution with a mean and standard deviation as per the estimates of Bletsa and Suchard was specified as an informative prior on the mean clock rate parameter [51]. For these data sets, the root height was also constrained to the 95% highest posterior density (HPD) interval of the estimates reported by Bletsa and Suchard [51]. For the other data sets, an uninformative prior was specified on the mean clock rate parameter, meaning that their evolutionary rate estimates were informed by the amounts of evolution that accumulated over the sampling time intervals. Moreover, for A2, the root height was constrained to be younger than that of subtype A’s most recent common ancestor (MRCA) [51]. When the exact date of sampling was not known (e.g., when only information on the month or year of sampling was available), the sampling date was estimated within the bounds of the known sampling time interval [52]. When the sampling date was unknown, it was estimated using a normal distribution with an offset equal to the difference between the data set’s most recent sampling date and the earliest date mentioned in the sample’s GenBank record. The mean and standard deviation of the normal distribution were based on the difference between the collection date and the oldest date reported in the GenBank record for a collection of sequences with a known sampling date [Pierard et al., unpublished].

Reconstructions of the virus spread relied on an asymmetric discrete phylogeographic model that incorporated a model averaging procedure (the Bayesian stochastic search variable selection procedure) to identify the subset of migration flows that adequately explain the diffusion process [53,54]. The expected number and timing of transitions between locations were estimated using stochastic mapping techniques [55]. The sampling location was set to Cyprus or the reported continent of infection. Sequences with an ambiguous location of sampling were accommodated using ambiguity coding in the phylogeographic approach [56].

The convergence and mixing properties of the Markov Chain Monte Carlo analyses were visually inspected using Tracer v1.7.1 [57]. Posterior tree distributions were summarized in the form of maximum clade credibility (MCC) trees and visualized using FigTree v.1.4.4 (http://tree.bio.ed.ac.uk/software/figtree/) (accessed on 12 February 2024).

#### 2.7.3. Transmission Cluster Identification

We implemented an update of a previously used R script to identify infection chains from a particular location using a combination of geographic and cluster root node support criteria [58]. For this, we capitalized on various R packages (ape v.5.6.2, phytools v.1.2.0, treeio v.1.20.2, dplyr v.1.0.10, and lubridate v.1.9.0) [41,59,60,61,62,63]. Specifically, starting from an MCC summary tree, we defined Cyprus-specific transmission clusters as clades with a size of two or more (i) with posterior root node support of ≥90% and (ii) for which the constituent lineages were inferred to have resided in Cyprus for at least 90% of the total time spanned by the cluster.

## 3. Results

In this prospective molecular epidemiology study examining the transmission dynamics within the HIV-1 epidemic in Cyprus, 313 consenting individuals diagnosed with HIV-1 and meeting the inclusion criteria were enrolled between 9 March 2017 and 14 October 2021. This cohort represented 60.4% of the total cumulative number of newly diagnosed individuals between 2017 and 2021 as reported by the European Centre for Disease Prevention and Control (ECDC), with a consent rate of 93.4% [10]. Nucleotide sequences spanning the HIV-1 *pol* region were successfully amplified and sequenced for 305 out of the 313 samples, with the remaining eight samples yielding negative results upon RT-PCR amplification. It is noteworthy that eight out of 305 PCR-positive samples were retrospectively reported to have an HIV-1 viral load below 10^3^ RNA copies/mL of plasma. Despite not meeting the inclusion criteria for the viral load, these samples were included for posterity purposes. The clinical, epidemiological, behavioral, and demographic data of the study participants are shown in Table S1 of the Supplementary Material. In-depth statistical analyses were performed on the acquired clinical, epidemiological, behavioral, and demographic information of the participating individuals and were subjected to subsequent cross-analysis with clustering phenomena. Accordingly, Table 3 summarizes the results of the statistical analyses.

### 3.1. The Cypriot HIV-1 Epidemic Predominantly Consists of Young to Middle-Aged Cypriot Males Residing in Nicosia, Who Reported MSM/HBC as a Risk Factor 

**Figure 1:**
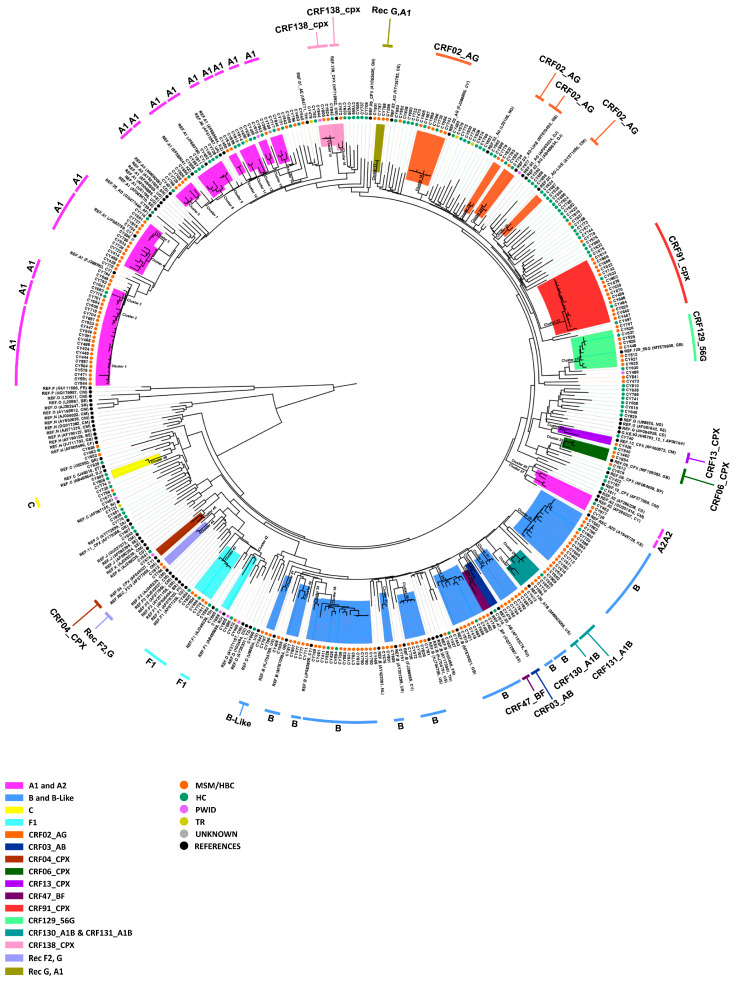
Maximum likelihood (ML) phylogenetic tree analyses of 305 HIV-1 *pol* region nucleotide sequences sampled between 9 March 2017 and 14 October 2021 in Cyprus. The nucleotide sequences were isolated from 305 people with HIV-1 (PWH) and residing in Cyprus at the time of sampling. The phylogenetic analyses were conducted against a comprehensive reference data set encompassing all known HIV-1 group M subtypes (A, B, C, D, F, G, H, J, K, and L) and circulating recombinant forms (CRFs) (RIP Alignment 2020) sourced from the Los Alamos HIV Sequence Database (http://www.hiv.lanl.gov (accessed on 12 February 2024)). Additionally, the reference data set was augmented through Basic Local Alignment Search Tool (BLAST) analyses using the HIV BLAST tool available through the Los Alamos HIV Sequence Database (www.hiv.lanl.gov/content/sequence/BASIC_BLAST/basic_blast.html (accessed on 12 February 2024)). The molecular clusters were defined based on previously established parameters of a genetic distance threshold of 0.045 and a bootstrap support threshold of 70%. The colored circles at the tips of the branches denote the respective risk factor and reference sequence associated with each HIV-1 subtype and CRF. Moreover, the identified HIV-1 molecular clusters are distinctly highlighted and color-coded at the periphery of the phylogenetic tree, in accordance with the HIV-1 genotypic subtypes determined by the REGA HIV-1 subtyping tool, version 3.0 (REGA 3.0) [31]. Each designated subtype is labeled adjacent to its corresponding HIV-1 molecular cluster. The acronyms used in the figure are as follows: MSM, men who have sex with men; HBC, homo-/bisexual contact; HC, heterosexual contact; PWID, people who inject drugs; TR, blood transfusion.

The demographic breakdown revealed that 78.69% of the cohort were male (*n* = 240), with the remaining 21.31% being female (*n* = 65) (Table 3). Additionally, the average age of the cohort was 37.8 years, with a median age of 35 years and an interquartile range (IQR) spanning from 29 to 44 years. Regarding risk factors, the majority, comprising 57.05% of the cohort, reported their risk factor as either MSM or HBC (*n* = 174), while 37.38% attributed their infection to HC (*n* = 114). A smaller proportion, 2.95%, identified as people who inject drugs (PWID) (*n* = 9), while 1.64% contracted the virus through blood transfusion (TR) (*n* = 5). Figure 1 shows an ML phylogenetic tree of all sequences utilized in this study, where the colored circles at the tips of branches denote the respective risk factor for each sample, illustrating the risk factor distribution among the cohort. The phylogenetic tree is supported by strong branching support values. It is important to note that the major clades received consistently high bootstrap values, indicating robust statistical support. The high bootstrap support across these key nodes reinforces the reliability of the inferred genetic evolutionary relationships.

In terms of geographical distribution, 86.89% of the cohort resided in urban areas (*n* = 265), indicating the predominance of urban settings in HIV-1 transmission within Cyprus. Further analysis of the cities of residence elucidated distinct patterns within the urban landscape. The capital city, Nicosia, hosted the largest proportion of PWH at 42.62% (*n* = 130), followed by Limassol at 22.62% (*n* = 69), Larnaca at 18.36% (*n* = 56), Pafos at 10.82% (*n* = 33), and Famagusta at 5.57% (*n* = 17).

In addition to the geographical distribution within Cyprus, this study also examined the countries of origin of the participating individuals, revealing the diverse origins contributing to the HIV-1 epidemic. Of the cohort, 47.54% hailed from Cyprus (*n* = 145), indicating a significant portion of local involvement in the epidemic. Notably, 52.46% of individuals reported other countries as their origins (*n* = 160), indicating a substantial proportion of foreign-born PWH within the cohort. 

Further analysis based on the regions of origin provided additional insights. Within Cyprus, as described previously, 52.46% of individuals originated from other countries (*n* = 160), with individuals from Sub-Saharan Africa constituting 26.23% (*n* = 80), highlighting the importance of immigration patterns in shaping the demographic profile of the HIV-1-affected population. Moreover, individuals from Western and Central Europe accounted for 17.05% (*n* = 52), reflecting the influence of migration from neighboring regions. Eastern Europe contributed 3.93% of the cohort (*n* = 12), and individuals from other regions constituted 5.25% (*n* = 16). Notably, 56.07% of individuals reported acquiring an HIV-1 infection within Cyprus (*n* = 171), whereas 43.93% attributed their infection to other countries (*n* = 134), underscoring the influence of both local and international factors in driving the epidemic.

Additionally, various other factors were examined as part of the analysis, the details of which can be found in Table 3. Briefly, the investigation focused on the infection status within the cohort, revealing that 96.39% were newly diagnosed with an HIV-1 infection and 3.61% had a chronic HIV-1 infection but all were reportedly drug-naïve. The analysis further explored key indicators of HIV-1 progression within the cohort. The Log10 viral load, with a standard deviation, demonstrated a median of 4.7 (with an interquartile range of 4.3–5.2), shedding light on the viral replication dynamics among the cohort, as a viral load in this range typically indicates active viral replication, which is consistent with an untreated HIV infection. Additionally, the CD4 cell count distribution revealed a median of 453 (with an interquartile range of 208–654), providing insights into the immune status of the cohort. While the median CD4 count of 453 cells/µL indicated that many participants had moderate immune function, the distribution suggests a varied level of immune compromise among the study participants, highlighting the need for the timely initiation of antiretroviral therapy to preserve immune function. Notably, the majority of individuals, at 93.11%, did not exhibit AIDS-defining illnesses (*n* = 284), while 6.89% reported such conditions (*n* = 21).

### 3.2. Polyphyletic HIV-1 Epidemic of Cyprus Predominated by Subtypes A1, B, and CRF02_AG

The subtype distribution of HIV-1 within the studied population offers valuable insights into the genetic diversity of the virus and its transmission dynamics. Subtypes A1 and B emerged as the predominant strains, constituting 23.93% (*n* = 73) and 20.00% (*n* = 61) of cases, respectively, with CRF02_AG also a notable contributor at 14.43% (*n* = 44) (Table 3). Additionally, CRF91_cpx, subtype C, subtype F1, and CRF129_56G were identified, each comprising 4.92% (*n* = 15), 4.26% (*n* = 13), 4.26% (*n* = 13), and 2.62% (*n* = 8) of cases, while subtype G and subtype A2 were observed at lower frequencies, representing 2.30% (*n* = 7) and 0.98% (*n* = 3) of cases, respectively. Notably, a substantial proportion of 22.30% fell under the category of “other” (*n* = 68), which represented other subtypes, indicating the presence of various recombinant forms and minor subtypes, where the majority were determined as uncharacterized recombinant forms (Figure 1). The phylogenetic tree in Figure 1 demonstrates the formation of distinct clades, suggesting the presence of well-defined genetic lineages. With numerous subtypes, CRFs, and uncharacterized recombinants in the Cypriot HIV-1 epidemic, 46 molecular clusters were identified in this phylogeny, 23 of which were pairs—14 for subtype A1, 10 for subtype B, 4 for CRF02_AG, and 11 for other CRFs—suggesting ongoing transmission chains within the population. 

### 3.3. Two Distinct HIV-1 Sub-Epidemics of Cyprus: Subtypes A1 and B

**Figure 2:**
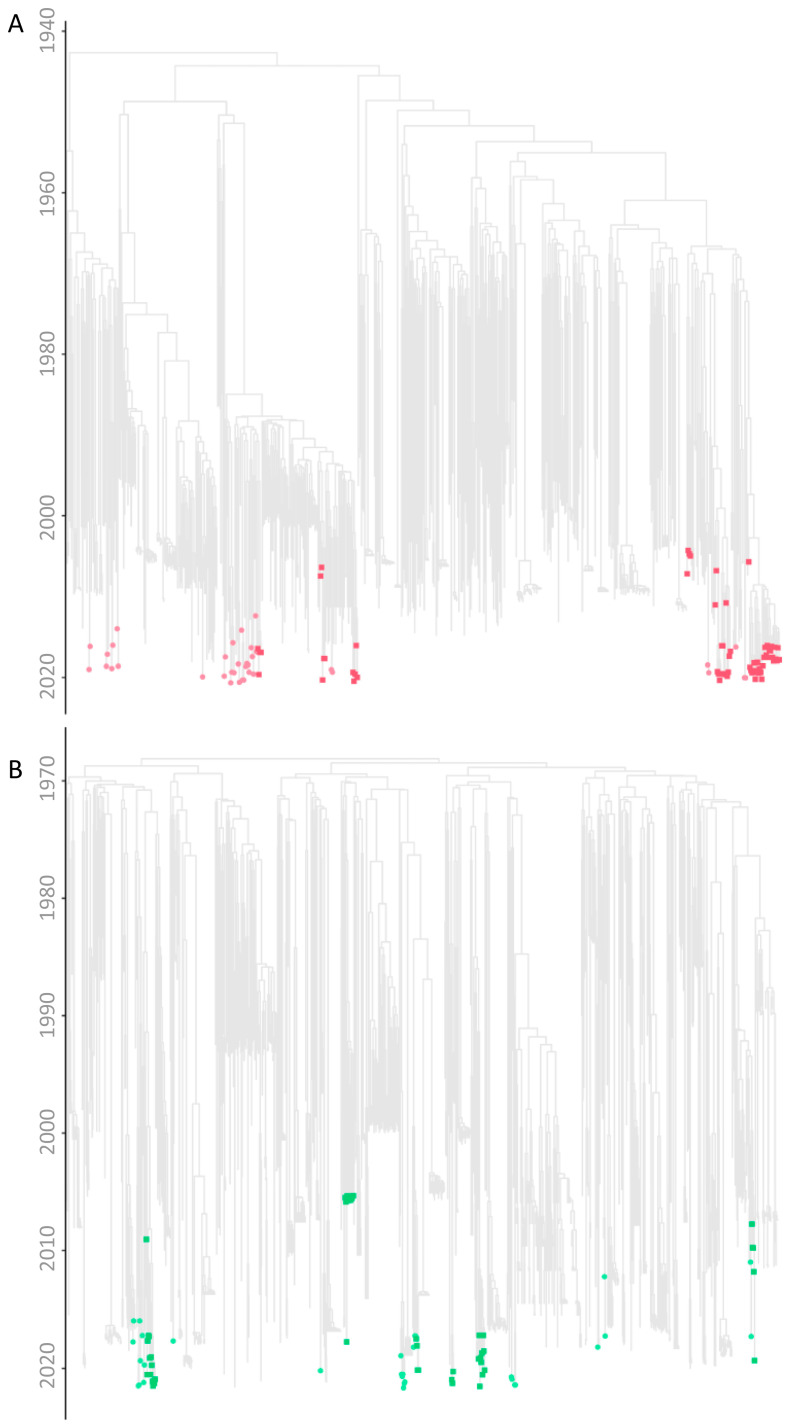

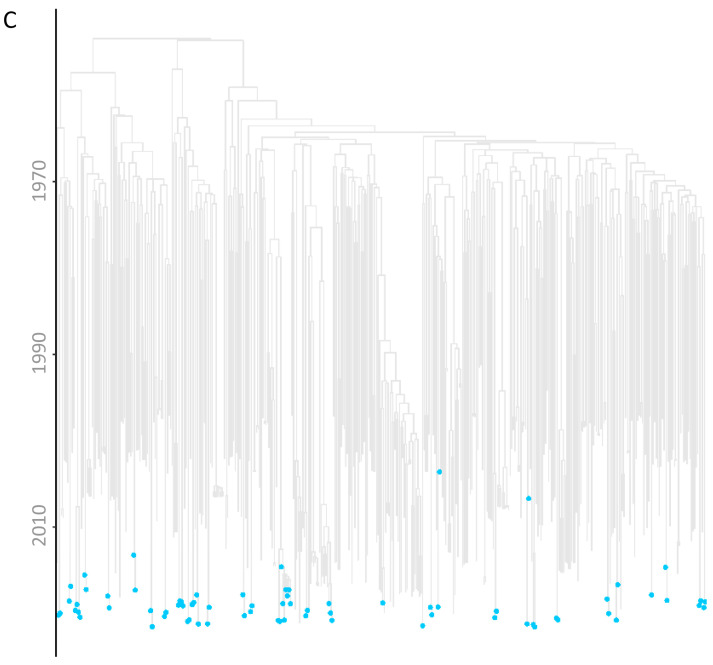
Time-scaled migration history derived from data sets utilized to reconstruct the spread history of (**A**) subtype A1, (**B**) subtype B, and (**C**) CRF02_AG. The gray tips correspond to reference sequences obtained from NCBI GenBank (accessed on 22 February 2024) [42]. Sequences that are part of a transmission cluster are indicated by dark pink squares (subtype A1) and dark green squares (subtype B) and cohort sequences that are not part of a transmission cluster by light pink circles (subtype A1), light green circles (subtype B) and blue circles (CRF02_AG).

**Figure 3:**
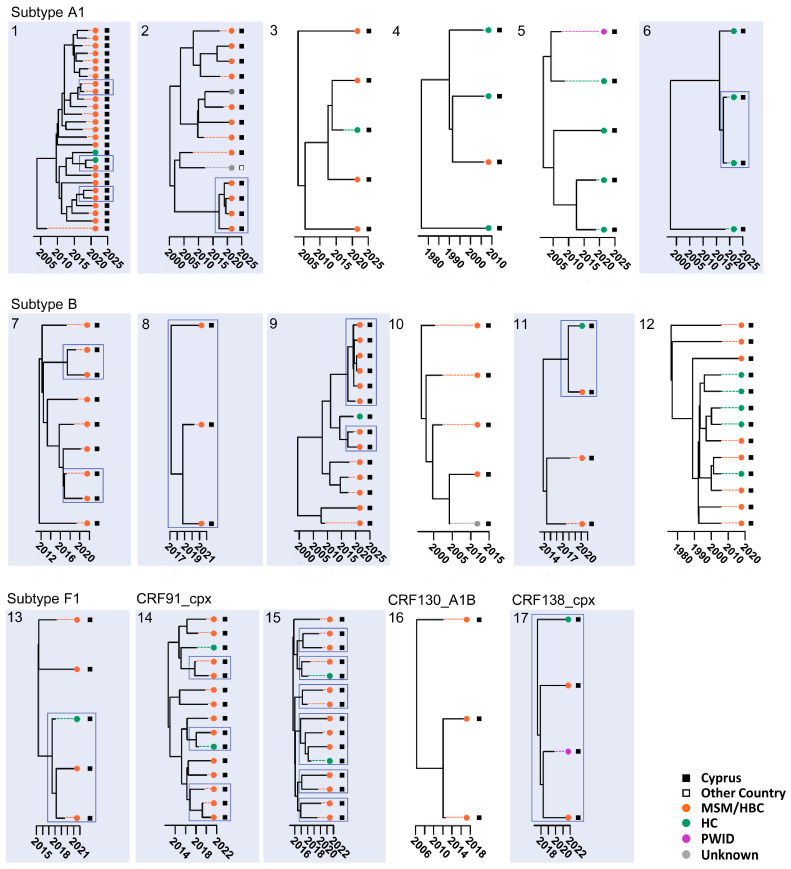
Identification of transmission clusters (TCs). The figure illustrates the identification of TCs based on stringent criteria, with a posterior support threshold of 90% and a minimum proportionate time spent in trait of 90%. Based on these criteria, sixteen distinct TCs have been discerned in this analysis: six attributed to subtype A1 (clusters 1–6), six to subtype B (clusters 7–12), and one each for subtypes F1 (cluster 13), CRF91_cpx (clusters 14–15), CRF130_A1B (cluster 16), and CRF138_cpx (cluster 17). Notably, the examination of recombinant strains entailed the separate analysis of non-recombinant regions. This approach led to the detection of two transmission clusters within the CRF02_AG (cluster 14) and subtype G (cluster 15) regions of the CRF91_cpx strain. Each TC is systematically numbered based on the associated HIV-1 subtype, with further categorization reflecting the decreasing prevalence of men who have sex with men (MSM) within the clusters of each subtype. Active TCs are distinctly highlighted in light blue, while clusters exhibiting ongoing activity within the past five years are delineated by dark blue rectangles, providing a temporal context for the depth of each cluster. The visual representation is enriched by the incorporation of colored circles at the tree tips, denoting the risk group, while squares symbolize the country of sampling (refer to the color code at the bottom of the figure). The acronyms used in the figure are as follows: MSM, men who have sex with men; HBC, homo-/bisexual contact; HC, heterosexual contact; PWID, people who inject drugs.

The time-scaled migration histories were reconstructed using the most prevalent strains identified in the studied cohort. The MCC trees in Figure 2 show the spread history of the three most predominant strains of the Cypriot epidemic, subtypes A1, B, and CRF02_AG. In the subtype A1 MCC tree, there were several well-supported Cypriot clades, as represented by the aggregation of the Cypriot taxa, indicating continuous inland transmission. Within these clades, six transmission clusters were identified, with posterior support of 90%, and, within a clade, we inferred the ancestral state to be Cyprus for at least 90% of the time represented by the clade (Figure 3). Five of these six transmission clusters were specific to Cyprus, demonstrating strong indications of local transmission, while one transmission cluster had a sequence isolated in the United Kingdom (UK). Each of the transmission clusters had 27, 14, 5, 4, 5, and 4 sequences, respectively, corresponding to transmission clusters 1–6, as depicted in Figure 3.

Similarly, in the subtype B MCC tree, there were well-supported Cypriot clades, as represented by the aggregation of the Cypriot taxa, although the sporadic topology of the Cypriot taxa was also observed, indicating both inland transmission and isolated importation events from other countries. Based on the above-described parameters, six transmission clusters were identified within the well-supported clades, all of which were specific to Cyprus, suggesting local transmission. Each of the transmission clusters had 9, 3, 14, 5, 4, and 13 sequences, respectively, corresponding to transmission clusters 7–12, as illustrated in Figure 3. In contrast to the subtype A1 and B migration histories, in the CRF02_AG MCC tree, there was no Cypriot clade formation. The sporadic topology of the Cypriot taxa on the CRF02_AG MCC tree hinted towards isolated importation events as a result of the influx of CRF02_AG infections. Accordingly, no transmission clusters of CRF02_AG were identified.

Additionally, one transmission cluster was identified for each of subtypes F1, CRF91_cpx, CRF130_A1B, and CRF138_cpx, corresponding to transmission clusters 13–17. It is important to acknowledge that a phylogenetic bifurcating tree model primarily represents vertical evolution, wherein the branches split apart. Conversely, recombination, a form of horizontal evolution, involves the merging of branches and cannot be accurately depicted by a phylogenetic bifurcating tree. As such, for the investigation of the time-scaled migration histories of recombinant strains, non-recombinant regions were analyzed separately. Consequently, for CRF02_AG and the G regions of CRF91_cpx, different evolutionary histories—as indicated by the differences in the branching structure in clusters 14 and 15—were identified prior to forming into a CRF (Figure 3).

### 3.4. Subtype A1 Sub-Epidemic Driven Mostly by Young MSM/HBC and HC Cases, Who Reported Contracting the Virus in Cyprus, and Subtype B Sub-Epidemic Driven Mostly by Middle-Aged and Older Cypriot MSM/HBC Cases, Who Reported Contracting the Virus in Cyprus

Analyzing the clustering phenomena revealed that, among individuals within the transmission clusters, subtype A1 predominated, comprising 46.67% (*n* = 49) of cases, followed by subtype B at 29.52% (*n* = 31), CRF91_cpx at 14.29% (*n* = 15), and subtype F1 at 4.76% (*n* = 5) (Table 3 and Figure 3). Notably, CRF02_AG, subtype C, CRF129_56G, subtype G, and subtype A2 were absent within the transmission clusters. 

**Figure 4:**
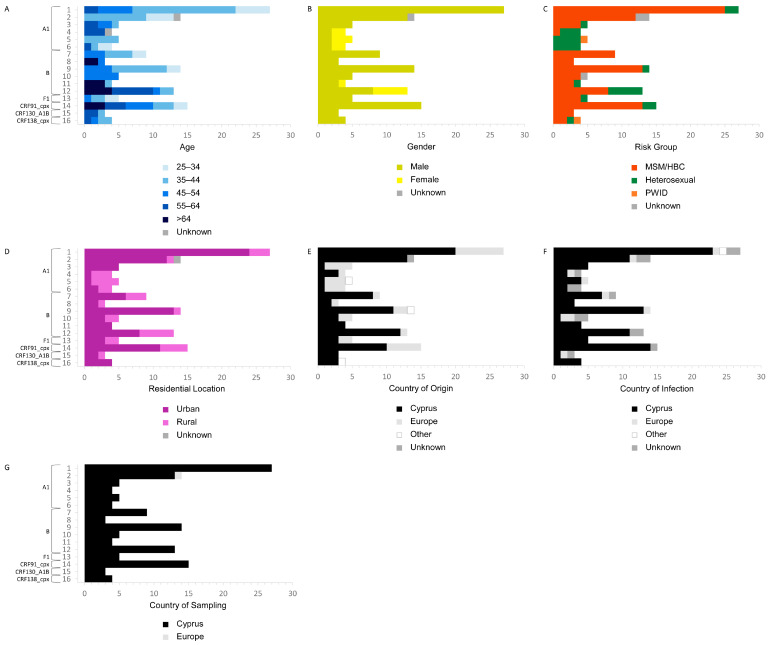
A comprehensive depiction of the epidemiological and demographic attributes associated with the identified transmission clusters (TCs). Within each TC, detailed insights are offered into the epidemiological information, including the (**A**) age distribution, (**B**) gender composition, (**C**) risk group affiliations, and demographic features, including the (**D**) residential location, (**E**) country of origin, (**F**) country of infection, and (**G**) country of sampling. The color coding is described below each graphical scheme. The acronyms used in the figure are as follows: MSM, men who have sex with men; HBC, homo-/bisexual contact; PWID, people who inject drugs.

Six transmission clusters were identified for subtype A1, comprising a total of 59 sequences (Figure 3). Among these, 71.19% were individuals aged between 25 and 44 (*n* = 42), with the most common age group being 35 to 44 (*n* = 31) (Figure 4). The age range for the A1 transmission clusters spanned from 26 to 62 years. Similarly, six transmission clusters were identified for subtype B, totaling 48 sequences. Of these, 60.42% were aged 45 and above (*n* = 29), while the remaining 39.58% fell between the ages of 25 and 44 (*n* = 19). The age range for B transmission clusters ranged from 30 to 83 years.

In the entire study cohort, 61.64% of individuals exhibiting subtype A1 reported their risk factor as MSM or HBC (*n* = 45), while 32.88% reported HC (*n* = 24). Specifically, the analysis of the risk factors for the A1 transmission clusters showed that 71.19% reported their risk factor as MSM or HBC (*n* = 42), while 23.73% reported HC (*n* = 14) (Figure 4). Conversely, the majority of individuals exhibiting subtype B in the overall study cohort reported their risk factor as MSM or HBC, accounting for 91.80% (*n* = 56). Similarly, the examination of the risk factors for B transmission clusters revealed that 83.33% reported their risk factor as MSM or HBC (*n* = 40). 

Further analyses revealed that, in the entire cohort, 54.79% of individuals exhibiting subtype A1 were native to Cyprus (*n* = 40), while the remaining 45.21% were from other countries (*n* = 33). Specifically, the investigation into the country of origin for the A1 transmission clusters showed that 66.10% reported Cyprus as their country of origin (*n* = 39), while 30.51% reported Europe (*n* = 18) (Figure 4). The examination of the country of infection for the entire study cohort revealed that 69.86% of those exhibiting subtype A1 reported acquiring the virus in Cyprus (*n* = 51), while, specifically for the A1 transmission clusters, this figure increased to 79.66% (*n* = 47) (Figure 4). Meanwhile, 77.05% of individuals exhibiting subtype B in the entire cohort were native to Cyprus (*n* = 47), with 83.61% reporting Cyprus as their country of infection (*n* = 51). Similarly, an analysis of the country of origin for the B transmission clusters showed that 83.33% originated from Cyprus (*n* = 40), while 81.25% reported Cyprus as their country of infection (*n* = 39). The major epidemiological characteristics of the subtype A1 and subtype B sub-epidemics are summarized in Table 4.

### 3.5. Limited Transmission of CRF02_AG in Cyprus Prodominantly among African HC Cases, Who Reported Contracting the Virus in Sub-Saharan Africa

Conversely, among individuals outside of the transmission clusters, the distribution of the subtypes differed, with CRF02_AG at 22.00% (*n* = 44), B at 15.00% (*n* = 30), A1 at 12.00% (*n* = 24), C at 6.50% (*n* = 13), F1 at 4.00% (*n* = 8), CRF129_56G at 4.00% (*n* = 8), G at 3.50% (*n* = 7), and A2 at 1.50% (*n* = 3) (Table 3). The *p*-value in terms of the transmission clusters and subtypes in the univariate analyses was calculated as <0.001, indicating a highly significant association between the HIV-1 subtype and clustering status. Moreover, further analyses into the third predominant strain on the island revealed that, in the entire cohort, 70.45% of the individuals affected by CRF02_AG originated from sub-Saharan Africa (*n* = 31) and 65.91% reported sub-Saharan Africa as their country of infection (*n* = 29). These findings align with the observed sporadic topology of the Cypriot taxa on the CRF02_AG MCC tree, supporting isolated importation events (Figure 2). Additionally, in contrast to subtypes A1 and B, the majority of the individuals exhibiting CRF02_AG reported their risk factor as HC, at 75.00% (*n* = 33). Interestingly, a significant proportion of the individuals exhibiting CRF02_AG were females (*n* = 18), representing 40.90%.

### 3.6. Male Cypriot MSM/HBC Cases, Residing in Nicosia and Limassol, Who Reported Contracting the Virus in Cyprus, Are Associated with the Onward Transmission of HIV-1

Notably, within the transmission clusters, an overwhelming 95.24% of the cohort were male (*n* = 100), compared to a low female proportion of 4.76% (*n* = 5), indicating the pronounced male predominance within these phylogenetically linked cohorts (Table 3 and Figure 4). In contrast, in cohorts outside of the transmission clusters, although males still formed the majority, with 70.00% (*n* = 140), the percentage of females was comparatively higher, comprising 30.00% of this subset (*n* = 60) (Table 3). The statistical analysis further validated these findings, revealing a significant association between gender and the clustering status, with a *p*-value of <0.001 in the univariate analyses. Significantly, within the entire study cohort, a substantial 90.77% of females originated from other countries (*n* = 59), with 63.08% specifically hailing from sub-Saharan Africa (*n* = 41). Notably, 96.92% of the females in the cohort were affected by non-B subtypes (*n* = 63), including CRF02_AG at 27.69% (*n* = 18), subtype A1 at 21.54% (*n* = 14), and a notable proportion of uncharacterized recombinants at 16.92% (*n* = 11). Conversely, among the males in the cohort, 24.58% were affected by subtype B (*n* = 59) and an equivalent percentage by subtype A1 (*n* = 59), while CRF02_AG accounted for 10.83% (*n* = 26).

The statistical analyses also illuminated the transmission dynamics, with a notable concentration of the cohort within the transmission clusters with MSM or HBC individuals, accounting for 82.86% (*n* = 87), compared to 15.24% attributed to HC (*n* = 16) (Table 3 and Figure 4). For the cohort outside of the transmission clusters, while MSM and HBC individuals still represented a significant portion at 43.50% (*n* = 87), they were represented by a notably lower percentage compared to within the transmission clusters (Table 3). HC constituted the highest percentage of the cohort outside of the transmission clusters, comprising 49.00% (*n* = 98). Other risk factors, such as PWID and TR, showed smaller proportions both within and outside of the transmission clusters. The statistical analysis underscored the significance of these findings, with a *p*-value of <0.001 indicating a strong association between the risk factors and clustering status in the univariate analyses. It is noteworthy that, in the entire cohort, 64.52% of MSM or HBC cases originated from Cyprus (*n* = 114), while 75.44% of HC cases originated from other countries (*n* = 86), primarily from sub-Saharan Africa, constituting 56.14% (*n* = 64). Furthermore, 32.76% of MSM or HBC cases were caused by subtype B (*n* = 57), followed by subtype A1 at 25.86% (*n* = 45). Conversely, among the HC cases in the cohort, 96.49% were caused by non-B subtypes (*n* = 110), including CRF02_AG at 29.82% (*n* = 34), subtype A1 at 21.05% (*n* = 24), and uncharacterized recombinants at 13.16% (*n* = 15).

Among the individuals within the transmission clusters, 34.29% resided in Nicosia (*n* = 36), followed by 31.43% in Limassol (*n* = 33), 16.19% in Larnaca (*n* = 17), 15.24% in Pafos (*n* = 16), and 2.86% in Famagusta (*n* = 3) (Table 3 and Figure 4). Conversely, for individuals outside of the transmission clusters, a higher proportion of 47.00% resided in Nicosia (*n* = 94), with smaller percentages in other cities: 19.50% in Larnaca (*n* = 39), 18.00% in Limassol (*n* = 36), 8.50% in Pafos (*n* = 17), and 7.00% in Famagusta (*n* = 14) (Table 3). The statistical analysis conducted on these observations yielded a *p*-value of 0.009, indicating a statistically significant association between the city of residence and clustering status. 

Among individuals within the transmission clusters, the majority, at 71.43%, were of Cypriot origin (*n* = 75), with the remainder originating from other countries at 28.57% (*n* = 30) (Table 3 and Figure 4). Conversely, among individuals outside of the transmission clusters, the proportion of individuals from Cyprus decreased to 35.00% (*n* = 70), while the majority hailed from other countries, at 65.00% (*n* = 130) (Table 3). The statistical analysis conducted on these observations yielded a *p*-value of <0.001, indicating a highly significant association between the country of origin and clustering status. In the entire cohort, 30.34% of individuals originating from Cyprus exhibited subtype B (*n* = 44), followed by subtype A1 at 27.59% (*n* = 40), with a relatively low proportion of CRF02_AG and uncharacterized recombinants, each accounting for 6.90% (*n* = 10). In contrast, among individuals originating from other countries in the cohort, 89.37% exhibited non-B subtypes (*n* = 143), including CRF02_AG at 21.25% (*n* = 34), subtype A1 at 20.63% (*n* = 33), and a relatively high proportion of uncharacterized recombinants at 16.88% (*n* = 27).

Analyzing the clustering phenomena in the broad context of the region of origin shed further light on these patterns. As previously described, among individuals within the transmission clusters, the majority, at 71.43%, originated from Cyprus (*n* = 75), followed by Western and Central Europe at 24.76% (*n* = 26), with minimal representation from other regions: 0.95% from sub-Saharan Africa (*n* = 1), 0.95% from Eastern Europe (*n* = 1), and 1.90% from other regions (*n* = 2) (Table 3). Conversely, among individuals outside of the transmission clusters, the proportion of individuals from Cyprus decreased to 35.00% (*n* = 70), while there was increased representation from sub-Saharan Africa, at 39.50% (*n* = 79), and other regions: 13.00% from Western and Central Europe (*n* = 26), 5.50% from Eastern Europe (*n* = 11), and 7.00% from other regions (*n* = 14). The statistical analysis conducted on these observations yielded a highly significant *p*-value of <0.001, indicating a strong association between the region of origin and clustering status. 

Notably, the analysis of the country of infection among the transmission clusters supported the transmission patterns indicated by the findings concerning the country of origin. Among individuals within the transmission clusters, the overwhelming majority at 89.52% reported acquiring the infection in Cyprus (*n* = 94), contrasting sharply with the minority, at 10.48%, who reported acquiring it in another country (*n* = 11) (Table 3 and Figure 4). Conversely, among individuals outside of the transmission clusters, a lower proportion of 38.50% reported acquiring the infection in Cyprus (*n* = 77), while the majority, at 61.50% (*n* = 123), reported acquiring it in other countries (Table 3). The highly significant *p*-value of <0.001 indicates a strong relationship between where the individuals contracted the virus and their clustering status within the cohort. The investigation of the entire cohort showed that 75.88% of individuals who contracted the virus in Cyprus reported their risk factor as MSM or HBC (*n* = 129). Among them, the majority exhibited subtype A1, representing 30.00% (*n* = 51), followed by subtype B, representing 27.65% (*n* = 47), and a relatively low proportion of uncharacterized recombinants, representing 7.06% (*n* = 12). In contrast, among individuals who contracted the virus in other countries in the cohort, 58.52% reported their risk factor as HC (*n* = 79). Individuals originating from other countries were predominantly affected by CRF02_AG, representing 25.29% (*n* = 34), followed by a significantly high proportion of uncharacterized recombinants, representing 18.52% (*n* = 25), and subtype A1, representing 16.30% (*n* = 22). Non-B subtypes collectively accounted for 89.63% (*n* = 131) of the individuals contracting the virus in other countries.

### 3.7. Subtype A1 and B Hotspots Driven by Cypriot MSM/HBC Cases Who Reported Contracting the Virus in Cyprus

**Figure 5:**
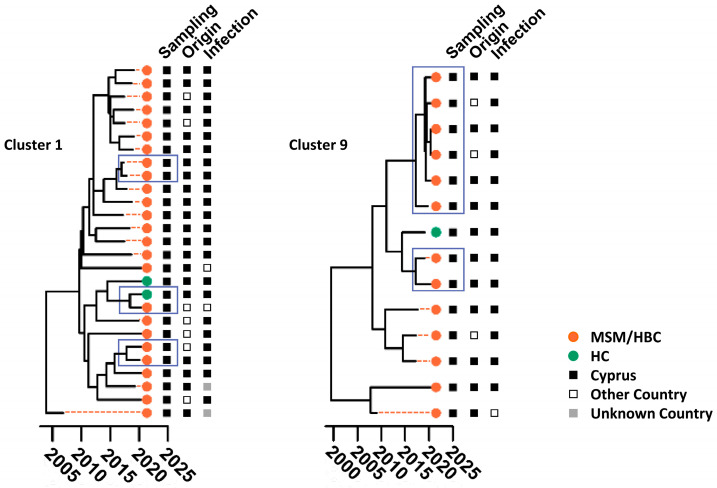
Transmission clusters (TCs) of subtypes A1 and B. The largest TCs associated with the two most prevalent HIV-1 subtypes in Cyprus, subtype A1 (Cluster 1) and subtype B (Cluster 9), are illustrated. Clusters exhibiting ongoing activity within the past five years are delineated by dark blue rectangles, providing a temporal context for the depth of each cluster. The visual representation is enriched by the incorporation of colored circles at the tree tips, denoting the risk group, while squares symbolize the country of sampling, country of origin, and country of infection, respectively (refer to the color code at the bottom of the figure). The acronyms used in the figure are as follows: MSM, men who have sex with men; HBC, homo-/bisexual contact; HC, heterosexual contact.

The demographic and behavioral profiles of the individuals in the largest A1 and B transmission clusters are delineated in Figure 5, providing insights into the characteristics of the individuals fueling the HIV-1 hotspots on the island. Both transmission clusters predominantly consisted of individuals of Cypriot origin, who reported acquiring the infection within Cyprus and identified their risk group as MSM or HBC. 

In detail, Cluster 1, the largest subtype A1 transmission cluster, comprised 27 individuals who reported their risk factors primarily as MSM or HBC (*n* = 25) and then HC (*n* = 2). These individuals mostly originated from Cyprus (*n* = 20), followed by Greece (*n* = 4), the UK (*n* = 1), Romania (*n* = 1), and Bulgaria (*n* = 1). In terms of the country of infection, the individuals mostly reported Cyprus (*n* = 23), followed by Greece (*n* = 1) and Israel (*n* = 1), while two cases were unknown. The largest subtype A1 transmission cluster (Cluster 1) was estimated to have originated in 2005, as determined by the Bayesian credible interval (BCI) with a 95% highest posterior density (HPD) ranging from 2004 to 2007. 

As for Cluster 9, the largest subtype B transmission cluster, it comprised 14 individuals who reported their risk factors primarily as MSM or HBC (*n* = 13) and then HC (*n* = 1). These individuals mostly originated from Cyprus (*n* = 11), followed by Indonesia (*n* = 1), Slovakia (*n* = 1), and Poland (*n* = 1). In terms of the country of infection, the individuals mostly reported Cyprus (*n* = 13) and then Greece (*n* = 1). Similarly, the largest subtype B transmission cluster (Cluster 9) was estimated to have originated in 1998, with a BCI of 95% HPD spanning from 1990 to 2005.

### 3.8. Time-Scaled Migration Histories

**Figure 6:**
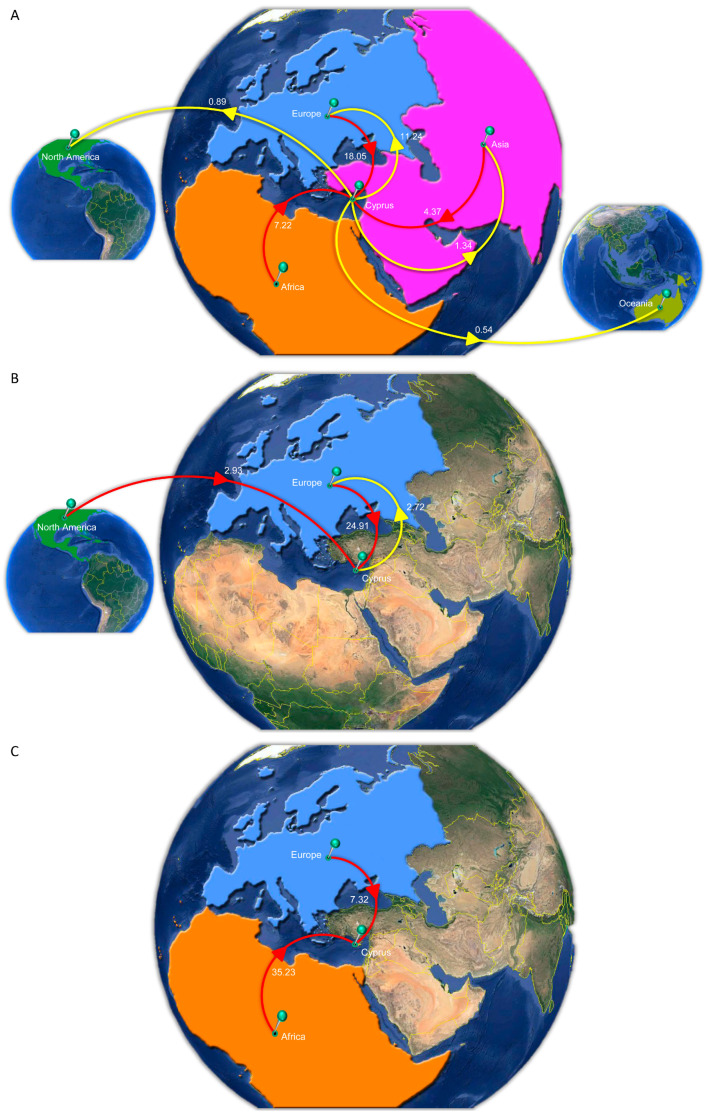
Cartographic representation of the transmission dynamics of HIV-1 (**A**) subtype A1, (**B**) subtype B, and (**C**) CRF02_AG between Cyprus and other continents. The geographic origins of these HIV-1 strains imported into Cyprus are depicted with red lines, while exports from Cyprus to other continents are illustrated with yellow lines. Continents serving as either “sources” or “sinks” for the transmission of these HIV-1 strains are highlighted and labeled accordingly. Additionally, the figure provides estimates of the average number of migration events between continents, offering insights into the intercontinental dissemination patterns. Map images courtesy of Google Earth Pro 7.3.2.5776 and 7.3.4.8642 (14 December 2015). Global view centered on North and South America (**left**), 5°49′53.21″ N 81°12′52.44″ W, Eye alt 9503.85 km. Europe (**middle**), 36°16′38.78″ N 36°07′29.71″ E, Eye alt 7949.12 km. South-Eastern Asia and Oceania (**right**), 1°14′19.88″ N 112°15′56.16″ E, Eye alt 11201.60 km. US Dept. of State Geographer, DATA SIO, NOAA, U.S. Navy, NGA, and GEBCO. Image Landsat/Copernicus. 2018 and 2023 © Google. https://www.google.com/earth/versions/#earth-pro (accessed on 10 April 2019 and 16 July 2023).

The time-scaled migration histories were reconstructed using the most prevalent strains identified within the studied cohort, as described previously (Figure 2). The locations of estimated importation and exportation events for the three most predominant strains of the Cypriot epidemic, subtypes A1, B, and CRF02_AG, are illustrated in Figure 6. Additionally, the overall data also including subtypes C, F1, and G are available in Table 5.

Subtype A1 emerged as the predominant HIV-1 strain within the Cypriot epidemic throughout the study duration. The initial import event, discerned through the BCI, was estimated to have occurred on 6 May 1966, with a 95% HPD interval spanning from 29 February 1960 to 17 January 1973. The ancestral location estimations of the first transmission event revealed that subtype A1 originated in Africa, with a posterior probability of 96%, while the remaining 4% of reconstructions of the ancestral location were estimated to be in Asia. The discrepancy between the importation and exportation events is evident in Table 5, where the total mean number of imports (29.64 Markov jumps, 100%) is more than double the total mean number of exports (14.00 Markov jumps, 100%). The ancestral locations of the first subtype A1 introduction into Cyprus were predominantly traced back to Africa with high support, which was in contrast with the majority of imports during the study period originating from Europe, accounting for 60.90% (18.05 Markov jumps) of the total imports (Figure 6A). Meanwhile, to a lesser extent, additional imports were attributed to Africa, accounting for 24.36% (7.22 Markov jumps), and Asia, accounting for 14.74% (4.37 Markov jumps) of the total imports, representing minor sources of subtype A1 in Cyprus. Similarly, Europe was the most frequent location to which Cyprus exported subtype A1, accounting for 80.29% (11.24 Markov jumps) of the total exports. While comparatively fewer in number, additional exports were linked to Asia, comprising 9.57% (1.34 Markov jumps); North America, comprising 6.36% (0.89 Markov jumps); and Oceania, comprising 3.86% (0.54 Markov jumps) of the total exports, accounting for minor sinks of subtype A1 from Cyprus. Overall, these findings reveal five continents involved in the migration of subtype A1 to and from Cyprus, with Africa identified as the primary origin and Europe as the predominant import source and export sink. Additionally, Africa and Asia emerged as other contributors to the transmission of subtype A1 to Cyprus, while Asia, North America, and Oceania were identified as other contributors to the transmission from Cyprus.

Subtype B was identified as the second most prevalent HIV-1 strain within the Cypriot epidemic during the study duration. Using the BCI, the initial import event was pinpointed to 27 October 1969, with a 95% HPD interval spanning from 24 July 1968 to 17 June 1971. The analysis of the ancestral locations of the first transmission event revealed North America as the definite origin of subtype B, with a posterior probability of 100%. The disparity between the importation and exportation events is evident in Table 5, where the total mean number of imports (27.83 Markov jumps, 100%) exceeds the total mean number of exports (2.72 Markov jumps, 100%) more than tenfold. Although the ancestral locations of the first introduction of subtype B into Cyprus were conclusively traced back to North America with a posterior probability of 100%, the majority of imports during the study period originated from Europe, accounting for 89.51% (24.91 Markov jumps) of the total imports (Figure 6B). Additional imports were attributed to North America, accounting for 10.53% (2.93 Markov jumps), representing a minority source of subtype B in Cyprus. Similarly, Europe was the sole destination to which Cyprus exported subtype B, comprising 100% (2.72 Markov jumps) of the total exports. Overall, these findings highlight the involvement of two continents in the migration of subtype B to and from Cyprus, with North America identified as the primary origin and Europe as the main import source and export sink. Additionally, North America emerged as another contributor to the transmission of subtype B from Cyprus.

CRF02_AG was determined as the third most common HIV-1 strain during the study period in Cyprus. The initial introduction of CRF02_AG to Cyprus, identified using the BCI, was traced back to September 2, 1966, with a 95% HPD interval spanning from 24 March 1960 to 26 December 1972. The ancestral location estimations of the first transmission event indicated Africa as the definite origin of CRF02_AG, with a posterior probability of 100%. Although no instances of exportation were detected, the recorded importation events are shown in Table 5, where the total mean number of imports (42.55 Markov jumps, 100%) can be observed. In line with the ancestral locations of the first introduction of CRF02_AG into Cyprus being conclusively from Africa, with a posterior probability of 100%, the majority of imports during the study period originated from Africa as well, accounting for 82.80% (35.23 Markov jumps) of the total imports (Figure 6C). A smaller proportion of imports were attributed to Europe, accounting for 17.20% (7.32 Markov jumps), representing a minority source of CRF02_AG in Cyprus. Overall, these results underscore the involvement of two continents in the migration of CRF02_AG to Cyprus, with Africa identified as both the primary origin and the main import source, while no exportation events were observed. Additionally, Europe emerged as another contributor to the transmission of CRF02_AG from Cyprus.

Further analysis of the minority subtypes identified in the Cypriot HIV-1 epidemic elucidated the transmission chains of subtypes C, F1, and G to and from Cyprus. Regarding subtype C, there was a total mean number of imports of 13.09 Markov jumps, with Africa being the primary import source, accounting for 11.36 Markov jumps. Additionally, a minor number of imports originated from Asia (1.03 Markov jumps) and Europe (0.70 Markov jumps). Concerning subtype F1, all importation events were traced back to Europe, totaling 11.34 Markov jumps. Similarly, for subtype G, all imports were from Africa, with a total mean number of imports of 7.23 Markov jumps. However, no exportation events were identified for these strains. These findings underscore the significance of international migration patterns in shaping the transmission dynamics of HIV-1 subtypes within Cyprus, with Africa and Europe serving as the primary sources for subtypes C, F1, and G.

### 3.9. HIV-1 Genotypic Drug Resistance

**Figure 7:**
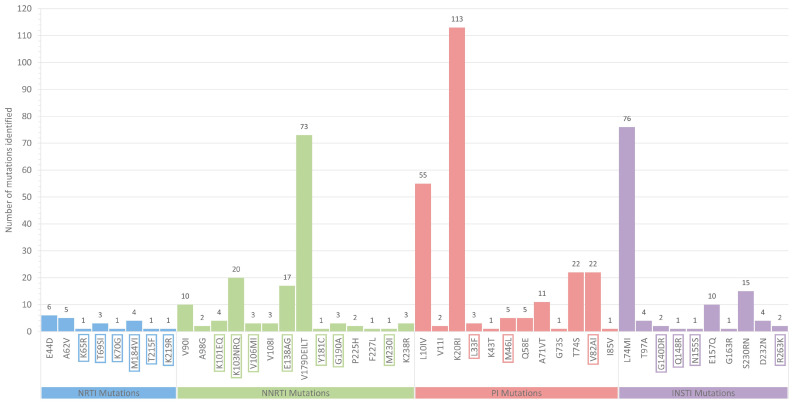
The overall prevalence of drug resistance-associated mutations identified among 305 HIV-1 *pol* region nucleotide sequences sampled between 9 March 2017 and 14 October 2021 in Cyprus. The graphical demonstration denotes the drug resistance-associated mutations identified within the *protease*, *reverse transcriptase*, and *integrase* (*PR*, *RT*, and *IN*) domains of HIV-1 group M subtypes, circulating recombinant forms (CRF), and recombinant strains, using the HIVdb Program of the Stanford University HIV Drug Resistance Database [32]. The *x*-axis delineates the drug resistance-associated mutations pertaining to the nucleoside reverse transcriptase inhibitors (NRTIs), non-nucleoside reverse transcriptase inhibitors (NNRTIs), protease inhibitors (PIs), and integrase strand transfer inhibitors (INSTIs). Each mutation identified against these classes of antiretroviral drugs is visually represented with distinct color coding: NRTI mutations are depicted in blue, NNRTI mutations in green, PI mutations in pink, and INSTI mutations in purple. The *y*-axis quantifies the frequency of occurrence for each mutation within the cohort, as denoted by the numerical values positioned atop each bar. Notably, mutations encased within rectangular boxes signify major drug resistance mutations, as defined by the Stanford University HIV Drug Resistance Database, while those not contained within these boxes are designated as accessory drug resistance mutations [64]. This classification scheme aids in discerning mutations of greater clinical significance from those with potentially lesser impacts on the drug resistance profiles.

The detailed overall prevalence of the drug resistance-associated mutations (DRMs) discovered among the 305 HIV-1 *pol* region nucleotide sequences among the study cohort is demonstrated in Figure 7. It is noteworthy that all 305 participating individuals were either newly diagnosed with or chronically affected by HIV-1 and were reportedly antiretroviral drug-naïve during sampling. Therefore, all DRMs identified in this study are attributed to transmitted drug resistance (TDR). Overall, 522 transmitted drug resistance-associated mutations (TDRMs) were identified within the cohort of this study. Of these 522 mutations, 18.39% were determined as major resistance mutations (*n* = 96), while the remaining 81.61% were determined as accessory resistance mutations (*n* = 426) linked to nucleoside reverse transcriptase inhibitors (NRTIs), non-nucleoside reverse transcriptase inhibitors (NNRTIs), protease inhibitors (PIs), and integrase strand transfer inhibitors (INSTIs). The drug resistance analysis revealed 22 NRTI mutations, 143 NNRTI mutations, 241 PI mutations, and 116 ISTI mutations, representing 4.21%, 27.39%, 46.17%, and 22.22% of all identified TDRMs, respectively. Further analysis showed that 11 of the NRTI, 49 of the NNRTI, 30 of the PI, and 6 of the INSTI mutations were determined as major resistance mutations, each representing 11.46%, 51.04%, 31.25%, and 6.25% of all major TDRMs identified among the cohort, respectively. Specifically, the determined major TDRMs included K65R (*n* = 1), T69SI (*n* = 3), K70G (*n* = 1), M184VI (*n* = 4), T215F (*n* = 1), and K219R (*n* = 1) linked to NRTIs; K101EQ (*n* = 4), K103NRQ (*n* = 20), V106MI (*n* = 3), E138AG (*n* = 17), Y181C (*n* = 1) G190A (*n* = 3), and M230I (*n* = 1) linked to NNRTIs; L33F (*n* = 3), M46L (*n* = 5), and V82AI (*n* = 22) linked to PIs; and G140DR (*n* = 2), Q148R (*n* = 1), N155S (*n* = 1), and R263K (*n* = 2) linked to INSTIs. The determined accessory TDRMs can be seen in Figure 7.

The total prevalence of resistance to HIV-1 antiretroviral drugs chosen by default on the HIVdb Program hosted by the Stanford University HIV Drug Resistance Database, among the newly diagnosed or chronically affected and reportedly antiretroviral drug-naïve study participants of the study, was 24.26% (*n* = 74). Of the 74 study participants presenting resistance to the NRTIs, NNRTIs, PIs, and INSTIs, 71 were newly diagnosed and reportedly drug-naïve, while the remaining were chronically affected by HIV-1 and reportedly drug-naïve. 

**Figure 8:**
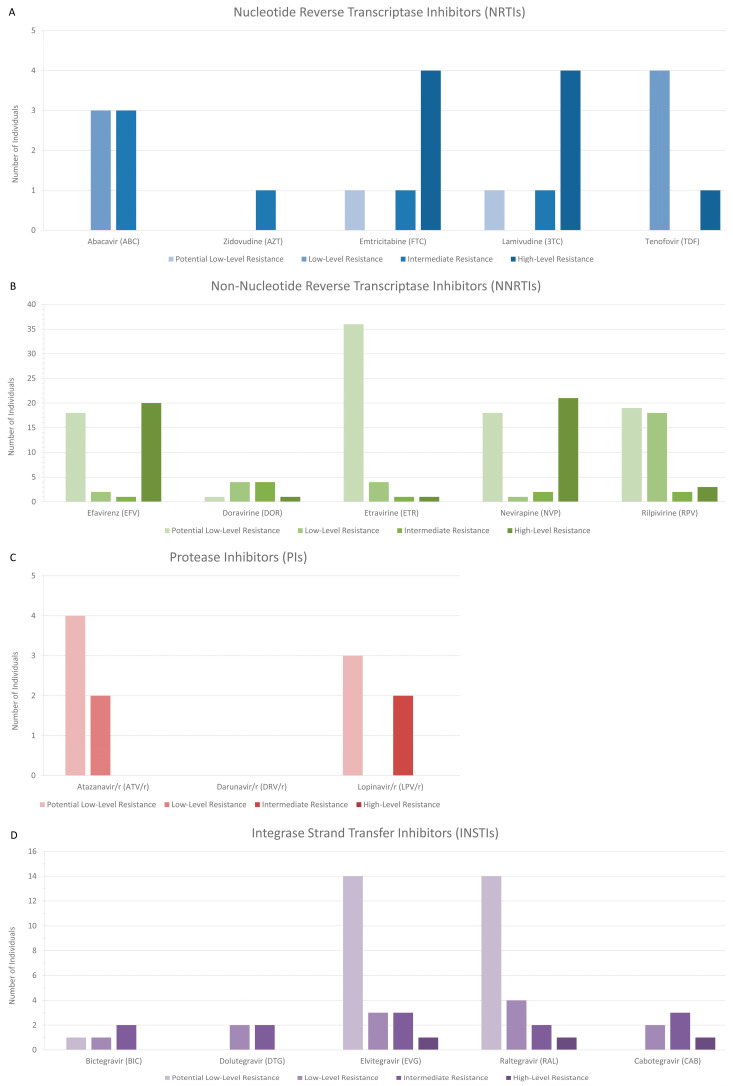
The overall prevalence of drug resistance levels against commercially available antiretroviral drugs identified among 305 HIV-1 *pol* region nucleotide sequences sampled between 9 March 2017 and 14 October 2021, in Cyprus. The graphical illustration delineates the extent of drug resistance against antiretroviral drugs categorized into (**A**) nucleoside reverse transcriptase inhibitors (NRTIs), (**B**) non-nucleoside reverse transcriptase inhibitors (NNRTIs), (**C**) protease inhibitors (PIs), and (**D**) integrase strand transfer inhibitors (INSTIs). The drug resistance levels are predicated on the drug resistance-associated mutations within the *protease*, *reverse transcriptase*, and *integrase* (*PR*, *RT*, and *IN*) domains of HIV-1 group M subtypes, circulating recombinant forms (CRF), and recombinant strains and were identified using the HIVdb Program of the Stanford University HIV Drug Resistance Database [32]. The four distinct levels of drug resistance, namely potential low-level resistance, low-level resistance, intermediate resistance, and high-level resistance, are visually represented with increasing intensity in color. Each level is color-coded to provide a clear representation of the escalating severity of the resistance. Across each graphical demonstration, the *x*-axis delineates the level of drug resistance against the commercially available antiretroviral drugs, while the *y*-axis quantifies the frequency of occurrence for each resistance level pertaining to each antiretroviral drug.

In total, we identified 268 instances of resistance spanning all drug classes, drugs, and resistance levels. Figure 8 illustrates the identified resistance in detail. Among NRTIs, 24 instances of resistance were observed, constituting 8.96% of the total. These included two potential low-level, seven low-level, six intermediate, and nine high-level resistance instances. Notably, three intermediate instances of resistance were found against ABC, and four high-level resistance instances each were identified against FTC and 3TC. Regarding NNRTIs, 177 resistance instances were identified, representing 66.04% of the total. These comprised 92 potential low-level, 29 low-level, 10 intermediate, and 46 high-level instances of resistance. Significant findings included four intermediate resistance instances against DOR and 20 and 21 high-level resistance instances against EFV and NVP, respectively. In the case of PIs, 11 resistance instances were noted, accounting for 4.10% of the total. These encompassed seven potential low-level, two low-level, and two intermediate-level instances of resistance. Unlike other classes, no high-level resistance instances were observed against PIs, although two intermediate resistance instances were found against LPV/r. Lastly, 56 instances of resistance against INSTIs were identified, constituting 20.90% of the total. Noteworthy findings included two intermediate resistance instances each against BIC, DTG, and RAL; three intermediate instances of resistance each against EVG and CAB; and one instance of high-level resistance each against EVG, RAL, and CAB. Additionally, notable degrees of lower-level resistance were observed against various drugs within the NNRTI and INSTI drug classes. Noticeably, 18 potential low-level resistance instances each were identified against EFV and NVP; 19 potential low-level resistance instances were identified against RPV; 36 potential low-level resistance instances were identified against ETR; and 18 low-level resistance instances were identified against RPV. Additionally, 14 potential low-level resistance instances each were revealed against EVG and RAL. Overall, our analysis provides comprehensive insights into the prevalence and distribution of TDRMs and various levels of resistance across different antiretroviral drug classes, aiding in the understanding and management of HIV-1 treatment within the Cypriot epidemic.

## 4. Discussion

Within this study, a thorough molecular epidemiological analysis was conducted, encompassing 305 HIV-1 sequences sampled from 305 respective PWH between 9 March 2017 and 14 October 2021 in Cyprus. Employing advanced statistical and bioinformatics techniques, the study delved deeply into the HIV-1 epidemic in Cyprus. Notably, the cohort examined in this study represents a substantial portion of all newly diagnosed cases reported by the ECDC between 2017 and 2021 in Cyprus, accounting for 60.4%, with a consent rate of 93.4% [10]. 

This investigation culminated in the characterization of Cyprus’s highly diverse HIV-1 epidemic, identifying the presence of various HIV-1 group M pure subtypes and CRFs, alongside numerous uncharacterized recombinant strains. The application of advanced bioinformatics analyses provided significant insights into the transmission dynamics within Cyprus and facilitated the mapping of transmission chains both originating from and extending to Cyprus, thereby elucidating the global transmission patterns of HIV-1 within the Cypriot context. Moreover, the study shed light on the TDR profile of the Cypriot HIV-1 epidemic, offering key insights into the efficacy of cART within the studied population. 

The demographic profile of the 305 PWH in Cyprus highlights several key observations. Primarily, the majority of the cohort consisted of young to middle-aged Cypriot males, particularly residing in urban areas, with Nicosia emerging as the primary epicenter of infection. This observation aligns with global trends, where urban settings often harbor higher rates of HIV-1 transmission due to the increased population density and accessibility to healthcare services such as HIV diagnostic testing [65,66]. The prevalence of MSM or HBC as the primary risk factor for HIV-1 acquisition underscores the importance of interventions within this demographic group to curb the transmission rates effectively. These findings indicate that the main epidemiological characteristics of the Cypriot epidemic persist, with the majority of cases being Cypriot male MSM or HBC cases who reported acquiring the infection within Cyprus and lived in Nicosia during sampling, which are consistent with the results of previous studies [24,25,26,27,28]. Additionally, our study demonstrates a further increase in MSM or HBC as the primary risk factor.

Furthermore, the significant representation of foreign-born individuals within the cohort reflects the influence of immigration patterns in shaping the HIV-1 epidemic in Cyprus. Substantial proportions of individuals originating from sub-Saharan Africa and Western and Central Europe indicate the diverse backgrounds contributing to the epidemic. The findings highlight a critical rise in the influx of individuals from sub-Saharan Africa, representing an 18.19% increase compared to a prospective molecular epidemiology study conducted from 1986 to 2012 in Cyprus [28]. Understanding the socio-cultural factors influencing migration and HIV-1 transmission among migrant populations is essential in designing tailored prevention and treatment strategies.

As a consequence of the geographic location of Cyprus at the intersection of three continents, Africa, Europe, and Asia, and the consequent import of numerous HIV-1 group M subtypes and CRFs from these continents, the HIV-1 epidemic on the island has become highly polyphyletic [28]. The monitoring of the HIV-1 genetic diversity within the Cypriot population, based on the HIV-1 *pol* region, reiterated the polyphyletic epidemic of Cyprus, with 21 HIV-1 group M pure subtypes and CRFs, predominantly characterized by subtype A1, subtype B, and CRF02_AG. Subtype C and F1 cases were also identified in the Cypriot epidemic, albeit in relatively small numbers. Additionally, investigations into the genotypic subtypes revealed that a significantly large proportion of the cohort (*n* = 72, 23.61%) consisted of uncharacterized recombinant strains, representing approximately double the prevalence identified in a prior study, which was 12% [27]. Notably, close to half of these uncharacterized recombinants (*n* = 33, 10.82%) were further characterized as five novel HIV-1 CRFs (CRF91_cpx, CRF129_56G, CRF130_A1B, CRF131_A1B, and CRF138_cpx) and two URFs, as described in previously published studies [18,19]. Such diversity suggests the complex interplay of factors shaping the epidemic landscape, including population mobility, migration, and the local transmission dynamics. This is also supported by nearly half of the cohort originating from Cyprus and the other half originating from other countries. 

The phylogenetic analysis presented in this study elucidates the formation of several distinct clades, indicating well-defined genetic lineages within the Cypriot HIV-1 epidemic. The circulation of diverse subtypes and recombinant forms underscores the complex and dynamic nature of the epidemic, with potential implications for treatment outcomes and the development of drug resistance. The identification of numerous molecular clusters further emphasizes the existence of ongoing transmission chains within the population, highlighting the need for surveillance and prevention efforts. The continued monitoring of HIV-1’s genetic diversity is crucial in understanding the evolving landscape of the epidemic. 

The characterization of the HIV-1 epidemic in Cyprus revealed the presence of two distinct sub-epidemics dominated by subtype A1 and subtype B, each displaying unique transmission dynamics and demographic profiles. The aggregation of the Cypriot taxa within the well-supported Cypriot clades on the subtype A1 and subtype B MCC trees indicates the continuous inland transmission of these subtypes. Although minimal, the sporadic topology of the Cypriot taxa was also observed on the subtype B MCC tree, indicating the additional influence of sporadic importation events from other countries feeding into the subtype B sub-epidemic. Moreover, within the identified transmission clusters, subtype A1 predominated, followed by subtype B, emphasizing localized transmission networks. The reconstruction of the time-scaled migration histories elucidated the spread of these predominant strains within the Cypriot epidemic, and their cross-analyses with the clinical, epidemiological, behavioral, and demographic data provided valuable insights into the underlying factors that drove their transmission. 

Specifically, the subtype A1 sub-epidemic was predominantly driven by young MSM or HBC cases, followed by HC cases, with a substantial proportion reporting the acquisition of the infection within Cyprus. Phylodynamic analysis identified several well-supported transmission clusters specific to Cyprus, indicating continued local transmission. In contrast, the subtype B sub-epidemic was characterized by middle-aged and older Cypriot MSM or HBC cases, also primarily contracting the virus within Cyprus. Similar to subtype A1, several transmission clusters specific to Cyprus were identified, underscoring the localized nature of transmission within the population. However, a notable distinction emerged in the country of origin within the transmission clusters. In the subtype A1 transmission clusters, a significant proportion originated from Europe (*n* = 18, 30.51%), contrasting with the subtype B clusters, where this figure was notably lower (*n* = 7, 14.58%). This discrepancy suggests the predominant influence of Cypriots in subtype B transmission, while subtype A1 transmission appears to be shaped by both Cypriots and Europeans residing on the island.

The diverse origins of PWH in Cyprus highlight the complex nature of the epidemic and its intersection with migration patterns. Localized transmission networks dominated by individuals from Cyprus contrast with sporadic infections driven by foreign-born individuals, indicating the interplay between local and international factors. However, while individuals from Cyprus were predominant contributors to the transmission clusters within the country, the considerable presence of individuals from Western and Central Europe within the transmission clusters suggests the notable influence of migration from neighboring regions on the dynamics of HIV transmission in Cyprus. This influx of individuals from Europe was observed to contribute to the formation of transmission clusters, as described above, highlighting the interconnectedness of regional epidemics, which suggests a potential role of cross-border transmission in fueling the HIV-1 epidemic in Cyprus. Factors such as migration, travel, tourism, and population mobility likely contribute to the spread of HIV within Cyprus, with the transmission dynamics influenced by interactions between individuals from diverse backgrounds. 

Despite being the third most prevalent strain within the epidemic, CRF02_AG displayed distinct transmission patterns compared to subtypes A1 and B. The exclusive sporadic topology of the Cypriot taxa on the CRF02_AG MCC tree suggests isolated importation events, highlighting the role of migration in introducing CRF02_AG into the population, rather than being locally transmitted on the island. The identification of no transmission clusters for CRF02_AG further supported the notion of the limited local transmission of this strain on the island. The findings of this study suggest that CRF02_AG was introduced into Cyprus through sporadic cases, rather than forming cohesive transmission networks within the local community. Further analyses showed that the vast majority of CRF02_AG cases originated from sub-Saharan Africa, which aligns with the significant representation of individuals originating from sub-Saharan Africa within the cohort, particularly among those outside of the transmission clusters. This observation is consistent with previous research indicating that CRF02_AG is prevalent in sub-Saharan Africa and is often associated with migration from this region to other parts of the world [67]. 

The relatively low representation of individuals from sub-Saharan Africa within the transmission clusters, in contrast to their high representation among the general cohort of the study, raises intriguing questions. This could be attributed to the limited mixing of the Cypriot population with the sub-Saharan African community, potentially due to factors such as social segregation, cultural differences, or geographic isolation. Additionally, individuals from sub-Saharan Africa, often seeking asylum and residing in rural regions or refugee camps, away from urban centers, where transmission clusters are more likely to occur, may have limited interaction with the broader population, potentially reducing their role as drivers of the Cypriot epidemic. 

Moreover, the presence of a relatively large CRF91_cpx transmission cluster, a newly characterized recombinant form found exclusively in Cyprus, suggests the local generation and spread of recombinants, which is commonly observed in polyphyletic HIV-1 epidemics. Notably, the high prevalence of “other” subtypes, predominantly uncharacterized recombinant forms, further highlights the dynamic nature of HIV-1’s evolution within the island, supporting presence of ongoing recombination events as well as the potential importation of diverse strains from other regions.

The predominant male composition of the general cohort, coupled with the even higher representation of males within phylogenetically linked cohorts, particularly among individuals within transmission clusters, underscores the disproportionate burden of HIV infection borne by males on the island. This male predominance within closely connected networks in Cyprus suggests a potentially higher susceptibility to HIV transmission, possibly due to engaging in high-risk behaviors. Conversely, the higher representation of females outside of the transmission clusters suggests a shift in gender dynamics, with females playing a more significant role in driving sporadic cases, possibly through different modes of transmission or within different social groups. This is supported by the observation that the females predominantly originated from sub-Saharan Africa and engaged in HC, which were also minimally represented within the transmission clusters, reinforcing the likelihood of their involvement in sporadic cases.

The wide age distribution of the cohort, spanning from younger adults to middle-aged individuals, emphasizes the importance of age-specific interventions for HIV prevention, testing, and treatment. Additionally, the large proportion of individuals attributing their infection to specific behaviors like MSM or HBC highlights the significance of sexual transmission routes in fueling the epidemic. Within the transmission clusters, this trend is even more pronounced, which suggests that networks of MSM or HBC individuals may serve as focal points for HIV transmission, emphasizing the importance of interventions within these communities. Conversely, sporadic infections among the Cypriot HIV-1 epidemic, particularly attributed to HC, indicate a shift in transmission dynamics beyond closely linked networks. 

The city residence analysis revealed Nicosia as a significant hub for HIV transmission, both within and outside of the transmission clusters. Nicosia, being the most populated and capital city of Cyprus, hosts populations from a broad range of backgrounds and communities, which might suggest its high percentage of representation among the phylogenetically distant cohort outside of the transmission clusters. However, for cohorts within the transmission clusters, there was a notable increase in the proportion of individuals residing in Limassol. These findings suggest dynamic patterns of HIV transmission within urban centers, with potential fluctuations in transmission dynamics between different cities over time.

This study also provides valuable insights into the time-scaled migration histories of HIV-1 subtypes within the Cypriot epidemic. Through Bayesian phylodynamic-based analysis, we reconstructed the spatiotemporal migration patterns of the most prevalent strains, focusing on subtypes A1, B, and CRF02_AG. These analyses revealed complex patterns of viral migration, importation, and exportation events over time.

With regard to the results, the initial import event of subtype A1, which was estimated to have occurred in the mid-1960s, was discovered as Africa, identified as the likely ancestral location. This finding underscores the role of international travel and migration in the introduction of HIV-1 into Cyprus, with the subsequent transmission dynamics shaping the local epidemic. The analysis of the importation and exportation events suggests a global migration network with contributions from multiple continents. Europe served as the predominant import source, although contributions from Africa and Asia were identified as well. While Europe also served as the predominant export sink, our findings also revealed minor exports to other continents. Subtype A1 exports to Asia, North America, and Oceania represented additional pathways of transmission, albeit in comparatively smaller numbers. These findings underscore the global interconnectedness of HIV-1 transmission networks, with multiple continents implicated in the dissemination of subtype A1.

The analysis of the migration histories of subtype B within the Cypriot epidemic exhibited distinct migration dynamics compared to subtype A1. The initial importation event of subtype B into Cyprus was estimated to have occurred in the late 1960s. The ancestral location of the first import was firmly traced back to North America. This underscores North America’s role as the primary origin of subtype B within the Cypriot epidemic. Interestingly, although North America served as the primary origin, it represented a minority source of subtype B in Cyprus during the studied period. The majority of imports during the studied period originated from Europe. This suggests ongoing transmission dynamics within Europe, contributing significantly to the prevalence of subtype B in Cyprus. Furthermore, the results revealed that Europe was the sole destination to which Cyprus exported subtype B. This highlights Europe’s role as the main export sink for subtype B from Cyprus, indicating predominant intracontinental transmission dynamics within Europe.

The examination of the time-scaled migration histories of CRF02_AG shed light on its transmission dynamics within the Cypriot HIV-1 epidemic. CRF02_AG exhibited distinct migration patterns compared to subtypes A1 and B. The initial introduction of CRF02_AG into Cyprus was estimated to have occurred in the mid-1960s. Africa was identified as the definite origin of the first importation event of CRF02_AG to Cyprus. Importantly, the analysis revealed that the majority of imports during the study period also originated from Africa, while Europe emerged as a minority contributor to the importation of CRF02_AG to Cyprus. This highlights Africa as the first and main import source of CRF02_AG in Cyprus, emphasizing the significance of transcontinental transmission dynamics between Africa and Cyprus. Furthermore, the ancestral origin of the CRF02_AG strain from Africa suggests that even instances of transmission originating in Europe are likely to be traced back to African sources, highlighting the enduring influence of African migration patterns on the epidemiology of this strain [68,69,70]. In contrast to subtypes A1 and B, no instances of exportation events were detected for CRF02_AG. This suggests a distinct pattern of migration for CRF02_AG, characterized by limited exportation from Cyprus to other regions.

The observed migration patterns inferred from the BEAST analysis strongly correlate with well-documented population-level migration in Cyprus, aligning with the island’s historical and current role as a crossroads between Europe, Asia, and Africa. Firstly, the significant influx of refugees and migrants from Africa is reflected in our analysis, particularly correlating with Figure 6A,C. These figures illustrate migration events that are consistent with the high levels of displacement from African countries, with many individuals seeking asylum or better living conditions in Cyprus due to its geographic proximity and relative political stability. Secondly, more recent migration patterns from Asia, primarily driven by economic factors, align with our findings in Figure 6A. Cyprus has become a key destination for individuals from various Asian countries, particularly those seeking employment opportunities in sectors such as construction, domestic work, and hospitality. The BEAST analysis captures these trends, indicating a discernible increase in migration from Asia in recent years. Additionally, Cyprus continues to attract a substantial number of individuals from Europe, either for tourism or as long-term residents seeking employment or retirement in the country. Figure 6A,B correspond with these migration patterns, highlighting the movement from various European countries to Cyprus. This trend is supported by the island’s appeal as a Mediterranean destination, offering favorable climate conditions and economic opportunities.

The analysis of the DRMs within the HIV-1 *pol* region among the study cohort revealed the significant prevalence of TDR. Despite all 305 individuals being reportedly antiretroviral drug-naïve at sampling, a total of 522 TDRMs were identified. Among these mutations, 18.39% were classified as major resistance mutations, with the remainder mostly being accessory mutations linked to various classes of antiretroviral drugs, including NRTIs, NNRTIs, PIs, and INSTIs. Notably, NNRTI mutations were the most prevalent, followed by PI mutations and INSTI mutations. Furthermore, a subset of these mutations was identified as major resistance mutations, indicating their potential impact on antiretroviral treatment outcomes. The presence of such mutations underscores the importance of the ongoing surveillance and monitoring of drug resistance patterns, particularly among treatment-naïve populations, to inform effective antiretroviral therapy strategies and minimize the emergence of drug resistance.

Among the drug resistance mutations identified, the V179E mutation clustered in eight out of nine samples within cluster 7, a subtype B transmission cluster shown in Figure 3. This mutation is linked to potential low-level resistance to several NNRTIs (EFV, ETR, NVP, and RPV). The lack of significant clustering among the observed drug resistance mutations suggests that these findings could be indicative of isolated importation events of resistant strains through international migration, rather than being generated through widespread inland transmission. This highlights the importance of continuous surveillance and the need for tailored public health strategies to address the potential for imported drug-resistant HIV strains.

The assessment of resistance to current HIV-1 antiretroviral drugs based on the identified TDRMs, among the newly diagnosed or chronically affected and reportedly antiretroviral drug-naïve individuals included in this study, revealed total prevalence of 24.26%. This encompassed resistance to NRTIs, NNRTIs, PIs, and INSTIs. Rigorous examination across these four key drug classes revealed resistance instances against specific drugs within each class. Among NRTIs, 24 resistance instances were observed, including potential low-level, low-level, intermediate, and high-level resistance, with notable findings against ABC, FTC, and 3TC. NNRTIs exhibited the highest prevalence of resistance, with 177 instances identified, including a significant number of intermediate and high-level resistance instances against EFV and NVP. PIs showed a lower prevalence of resistance, with significant findings against DRV/r and LPV/r. Additionally, INSTIs displayed notable intermediate and high-level resistance instances, particularly against EVG, RAL, and CAB. Moreover, lower-level resistance was observed across the NNRTI and INSTI drug classes, indicating the potential for further resistance development. The findings of the study highlight a substantial increase in transmitted drug resistance compared to previous studies, indicating a highly concerning trend in the efficacy of antiretroviral therapy within the studied population [24,26,27]. Although Cyprus has historically exhibited consistently low levels of TDR compared to Europe, the current prevalence of TDR in Cyprus is approximately twice that observed in the European region [71,72].

## 5. Conclusions

Through a comprehensive exploration of molecular epidemiology encompassing 305 HIV-1 sequences collected between 9 March 2017 and 14 October 2021, this study offers a comprehensive understanding of the HIV-1 epidemic in Cyprus. Leveraging advanced statistical and bioinformatics methodologies, the discoveries of the study have significantly contributed to our knowledge of the complex dynamics of HIV-1 transmission within the region. Notably, the substantial representation of newly diagnosed cases within the cohort, accounting for 60.4% of all cases reported by the ECDC between 2017 and 2021, underscores the relevance and significance of the findings presented here.

The characterization of Cyprus’s HIV-1 epidemic revealed a highly diverse landscape, comprising 21 HIV-1 group M pure subtypes and CRFs, alongside numerous uncharacterized recombinant strains. Among these, subtypes A1 and B emerged as the two most prevalent strains, followed by CRF02_AG. The identified diversity, coupled with insights into the transmission dynamics facilitated by advanced bioinformatics analyses, highlights the intricate interplay of factors driving the epidemic. Furthermore, the assessed high levels of TDR patterns underscore the importance of ongoing surveillance to inform effective antiretroviral therapy strategies.

The demographic profile of individuals involved in the onward transmission of HIV-1 in Cyprus highlights the disproportionate burden borne by young to middle-aged Cypriot males, particularly those belonging to the MSM community, who reported contracting the virus in Cyprus. The study also demonstrates the importance of the field of molecular epidemiology in understanding the socio-cultural factors that influence migration and HIV-1 transmission among migrant populations, particularly those originating from sub-Saharan Africa and Western and Central Europe. This understanding serves as a critical component in the design of tailored prevention and treatment strategies.

The analysis of the time-scaled migration histories sheds light on the global interconnectedness of the HIV-1 transmission networks, with five continents implicated in the dissemination of strains within Cyprus: Europe, Africa, Asia, North America, and Oceania. These findings underscore the need for coordinated efforts across borders to effectively address the challenges posed by HIV-1 transmission.

In conclusion, this study advances our comprehension of the HIV-1 epidemic dynamics in Cyprus. By providing valuable insights into the genetic diversity, transmission dynamics, demographic patterns, and drug resistance profiles, this study lays the groundwork for public health interventions aimed at curbing the spread of HIV-1 and improving patient outcomes in Cyprus and beyond.

## Figures and Tables

**Table 1:** Overview of the CRF-specific non-recombinant subregions that were used in the similarity searches for the data set’s compilation.

Circulating Recombinant Form (CRF)	Non-Recombinant Subregion	HXB2 Positions ^a^
CRF91_cpx	CRF02AG	2253–3034; 3485–5250
	G	3035–3484
CRF129_56G	CRF02	2253–2701; 3203–3332; 3832–4076; 4143–4427; 4812–5250
	B	2702–3202; 3333–3831; 4077–4142
CRF130_A1B	A1	2253–2284; 3535–4259; 4899–5250
	B	2285–3534; 4260–4898
CRF131_A1B	A1	2253–2284; 3827–4250; 4823–5250
	B	2285–3826; 4251–4822
CRF138_cpx	CRF22_01A1	2253–2061; 3024–4211; 4382–5250
	F2	2062–3023
	U	4212–4381

^a^ When the breakpoint was not exactly known, the midpoint of the breakpoint region was used to delineate the non-recombinant regions.

**Table 2:** Overview of the data set sizes.

Data Set	Number of Taxa	Number of Non-Cohort Taxa
A1	645	572
A2	79	76
B	1054	993
C	605	592
F1	265	252
G	151	144
CRF02_AG	392	348
CRF91_cpx	51 (CRF02_AG)	36
	90 (G)	75
CRF129_56G	58 (B)	50
	92 (CRF02_AG)	84
CRF130_A1B	5 (A1)	4
	22 (B)	21
CRF131_A1B	14 (A1)	10
	75 (B)	71
CRF138_cpx	47 (CRF22_01A1)	43
	48 (F2)	44
	52 (U)	48

**Table 3:** Statistical analysis of the clinical, epidemiological, behavioral, and demographic characteristics of the 305 people with HIV-1 (PWH) that were included in this study; 105 of these individuals were identified in the transmission clusters and 200 of these individuals were identified outside of the transmission clusters.

Variable	Total Cohort		Cohort in TCs ^a^		Cohort out of TCs ^b^		
	*n* = 305	%	*n* = 105	%	*n* = 200	%	*p*-Value ^c^
Gender ^1^							
Male	240	78.69	100	95.24	140	70.00	<0.001
Female	65	21.31	5	4.76	60	30.00	
Age							
Mean	37.8		39.4		36.9		0.088
Median, IQR ^d,2^	35 (29–44)		37 (30–45)		35 (28–43)		
Risk Factor ^e,1^							
MSM/HBC	174	57.05	87	82.86	87	43.50	<0.001
HC	114	37.38	16	15.24	98	49.00	
PWID	9	2.95	1	0.95	8	4.00	
TR	5	1.64	0	0.00	5	2.50	
Unknown	3	0.98	1	0.95	2	1.00	
Area of Residence ^1^							
Urban	265	86.89	88	83.81	177	88.50	0.33
Rural	40	13.11	17	16.19	23	11.50	
City of Residence ^1^							
Nicosia	130	42.62	36	34.29	94	47.00	0.009
Limassol	69	22.62	33	31.43	36	18.00	
Larnaca	56	18.36	17	16.19	39	19.50	
Pafos	33	10.82	16	15.24	17	8.50	
Famagusta	17	5.57	3	2.86	14	7.00	
Country of Origin ^1^							
Cyprus	145	47.54	75	71.43	70	35.00	<0.001
Other	160	52.46	30	28.57	130	65.00	
Region of Origin ^1^							
Cyprus	145	47.54	75	71.43	70	35.00	<0.001
Sub-Saharan Africa	80	26.23	1	0.95	79	39.50	
Western and Central Europe	52	17.05	26	24.76	26	13.00	
Eastern Europe	12	3.93	1	0.95	11	5.50	
Other	16	5.25	2	1.90	14	7.00	
Country of Infection ^1^							
Cyprus	171	56.07	94	89.52	77	38.50	<0.001
Other	134	43.93	11	10.48	123	61.50	
Pregnancy at Diagnosis ^1^ (*n* = 65) (Females only)							
No	58	89.23	4	80.00	54	90.00	0.45
Yes	7	10.77	1	20.00	6	10.00	
Infection Status ^1^							
Newly Diagnosed	294	96.39	99	94.29	195	97.50	0.27
Chronic	11	3.61	6	5.71	5	2.50	
Log10 Viral Load [SD] ^f,2^ (*n* = 301) (Unknown excluded)							
Median, IQR	4.7 (4.3–5.2)		4.6 (4.2–5.2)		4.7 (4.3–5.2)		0.69
CD4 Cell Count ^2^							
Median, IQR	453 (208–654)		497 (300–718)		436 (189–595)		0.018
CD4 Count Category ^1^							
<200	183	60.00	68	64.76	115	57.50	0.03
200–350	51	16.72	22	20.95	29	14.50	
>350	68	22.30	15	14.29	53	26.50	
Unknown	2	0.66	0	0.00	2	1.00	
AIDS-Defining Illness ^1^							
No	284	93.11	100	95.24	184	92.00	0.41
Yes	21	6.89	5	4.76	16	8.00	
HBsAg ^g,1^ (*n* = 303) (Unknown excluded)							
Negative	299	98.03	102	97.14	197	98.50	0.61
Positive	4	1.31	2	1.90	2	1.00	
Anti-HCV ^h,1^ (*n* = 302) (Unknown excluded)							
Negative	298	97.70	104	99.05	194	97.00	0.3
Positive	4	1.31	0	0.00	4	2.00	
Subtype ^1^							
A1	73	23.93	49	46.67	24	12.00	<0.001
B	61	20.00	31	29.52	30	15.00	
CRF02_AG	44	14.43	0	0.00	44	22.00	
CRF91_cpx	15	4.92	15	14.29	0	0.00	
C	13	4.26	0	0.00	13	6.50	
F1	13	4.26	5	4.76	8	4.00	
CRF129_56G	8	2.62	0	0.00	8	4.00	
G	7	2.30	0	0.00	7	3.50	
A2	3	0.98	0	0.00	3	1.50	
Other	68	22.30	NA ^i^		NA		

^a^ The population in the transmission clusters (TCs). ^b^ The population outside of the transmission clusters. ^c^ *p*-value indicates the probability for the univariate analysis. ^d^ IQR, interquartile range. ^e^ MSM, men who have sex with men; HBC, homo-/bisexual contact; HC, heterosexual contact; PWID, people who inject drugs; TR, blood transfusion. ^f^ SD, standard deviation. ^g^ HBsAg, surface antigen of hepatitis B virus. ^h^ Hepatitis C virus. ^i^ N/A, not available. ^1^ For each categorical variable, determinants for clustering were investigated using the chi-squared test or Fisher’s exact test. ^2^ For each continuous variable, determinants for clustering were investigated using a *t*-test.

**Table 4:** Major epidemiological characteristics of subtype A1 and subtype B sub-epidemics.

Variable	Subtype A1				Subtype B			
	Total Cohort		Cohort in TCs ^a^		Total Cohort		Cohort in TCs ^b^	
	*n* = 73	%	*n* = 59	%	*n* = 61	%	*n* = 48	%
Risk Factor								
MSM/HBC ^c^	45	61.64	42	71.19	56	91.80	40	83.33
HC	24	32.88	14	23.73	5	8.20	7	14.58
Country of Origin								
Cyprus	40	54.79	39	66.10	47	77.05	40	83.33
Other	33	45.21	20	33.90	14	22.95	8	16.67
Country of Infection								
Cyprus	51	69.86	47	79.66	51	83.61	39	81.25
Other	22	30.14	12	20.34	10	16.39	9	18.75

^a^ The population exhibiting subtype A1 in the transmission clusters (TCs). ^b^ The population exhibiting subtype B in the TCs. ^c^ MSM, men who have sex with men; HBC, homo-/bisexual contact; HC, heterosexual contact.

**Table 5:** The estimation of the migration events directed towards and originating from Cyprus.

Subtype ^a^	From ^b^	To ^c^	Average ^d^	Lower ^e^	Upper ^f^
A1	All ^g^	Cyprus	29.64	25	34
	Europe	Cyprus	18.05	13	22
	Africa	Cyprus	7.22	5	9
	Asia	Cyprus	4.37	4	5
	Cyprus	All	14.00	11	19
	Cyprus	Europe	11.24	9	15
	Cyprus	Asia	1.34	0	3
	Cyprus	North America	0.89	0	3
	Cyprus	Oceania	0.54	0	1
B	All	Cyprus	27.83	25	30
	Europe	Cyprus	24.91	17	30
	North America	Cyprus	2.93	0	8
	Cyprus	All	2.72	1	5
	Cyprus	Europe	2.72	1	5
CRF02_AG	All	Cyprus	42.55	41	44
	Africa	Cyprus	35.23	33	37
	Europe	Cyprus	7.32	6	9
C	All	Cyprus	13.09	13	14
	Africa	Cyprus	11.36	10	12
	Asia	Cyprus	1.03	1	1
	Europe	Cyprus	0.70	0	1
F1	All	Cyprus	11.34	10	12
	Europe	Cyprus	11.34	10	12
G	All	Cyprus	7.23	6	8
	Africa	Cyprus	7.23	6	8

^a^ The predominant HIV-1 genotypic subtypes observed in Cyprus from March 9 2017 to October 14 2021 were identified utilizing the REGA HIV-1 subtyping tool version 3.46. This tool is accessible at the following link: https://www.genomedetective.com/app/typingtool/hiv (accessed on 8 February 2024). ^b,c^ “From” and “To” designate the continents/subregions from which migration events originated or to which they were directed, respectively. Continent/subregion classifications are based on United Nations (UN) geographical subregions. ^d–f^ The average Markov jumps were determined by calculating the lower and upper bounds of the 95% highest posterior density (HPD) interval for migration events to and from Cyprus. ^g^ “All” denotes the amalgamation of all migration events.

## Data Availability

The HIV-1 *pol* region (2253–5096 in the HXB2 genome) nucleotide sequence data of the viral isolates generated in this study are openly available in GenBank at https://www.ncbi.nlm.nih.gov/genbank/ (accessed on 25 June 2024), and the accession numbers are ON989213–ON989244, ON989247–ON989271, ON989274–ON989279, ON989281–ON989297, ON989299–ON989306, ON989308–ON989316, ON989319–ON989328, ON989330–ON989341, PP909549–PP909716, OK584018, OK283056–OK283065, OP781327, OP781329, OP781330, and OP894080–OP894083. The molecular analysis executed in this study was performed on HIV-1 *pol* region sequences (2253–5250 in the HXB2 genome) that were 159 nucleotides longer than the sequences deposited in GenBank.

## Supplementary Materials

The following supporting information can be downloaded at: https://www.mdpi.com/article/10.3390/v16091449/s1, Table S1: Clinical, epidemiological, behavioral, and demographic information of the 305 individuals living with HIV-1 that were included in this study.
