# Supplementary material for: Mapping Transmission Dynamics and Drug Resistance Surveillance in the Cyprus HIV-1 Epidemic (2017–2021)
Source: Viruses. 2024 Sep 11;16(9):1449. doi: 10.3390/v16091449 (PMC11437465; doi:10.3390/v16091449)
Supplement: Supplementary file 1 [file viruses-16-01449-s001.zip › viruses-3100686-supplementary.pdf]

Table S1. Clinical, epidemiological, behavioral, and demographic information of the 305 individuals living with HIV-1 that were included in this study.

Patient [a]	Sex [b]	Age Group (years)	Sample Collection Date	Positive Test Date [c]	Country of origin [d]	Risk group [e]	CD4 (cells/mm^3)	Plasma HIV-1 RNA (copies x 10^3/ml)	Epidemiological Information [f]	GenBank Accession Numbers
CY391	M	30-39	03/17	08/16	Cyprus	HBC	631	2.01	Infected in Cyprus	ON989213
CY393	M	70-79	03/17	-/12	Egypt	HC/OHPC	37	5,320.00	Infected in Cameroon	ON989214
CY394	M	60-69	03/17	03/17	Cyprus	HC	17	134.00	Infected in Greece	ON989215
CY395	M	30-39	03/17	02/17	Russia	MSM	384	228.00	Infected in Russia	ON989216
CY396	M	60-69	03/17	01/17	Cyprus	MSM	1,093	8.91	Infected in Cyprus	ON989217
CY398	M	40-49	03/16	05/16	Cyprus	MSM	523	20.80	Infected in Cyprus	ON989218
CY399	M	40-49	03/16	01/17	Cyprus	HBC	1,093	350.00	Infected in Cyprus/UK	ON989219
CY401	M	40-49	03/17	04/15	Bulgaria	HC	726	29.30	Infected in Cyprus	ON989220
CY403	M	30-39	03/17	11/13	Cyprus	HBC	545	49.40	Infected in Cyprus	ON989221
CY405	M	40-49	03/17	09/17	Cyprus	HBC	452	50.00	Infected in Europe	ON989222
CY408	M	30-39	03/17	11/16	Cyprus	MSM	269	240.00	Infected in Cyprus	ON989223
CY409	M	40-49	03/17	01/17	Cyprus	MSM	420	98.80	N/A	ON989224
CY411	M	40-49	03/17	03/15	Cyprus	MSM	509	72.00	Infected in Cyprus	ON989225
CY413	M	50-59	03/17	10/13	Cyprus	HBC	1,216	25.00	Infected in Cyprus	ON989226
CY414	M	30-39	03/17	07/15	Cyprus	MSM	893	9.02	Infected in Cyprus	ON989227
CY418	M	40-49	03/17	10/14	Cyprus	HBC	706	8.14	Infected in Cyprus	ON989228
CY419	M	30-39	04/17	07/16	Cyprus	MSM	975	52.70	Infected in Cyprus/Greece	PP909549
CY421	M	40-49	04/17	01/13	Cyprus	HBC	646	20.80	Infected in Cyprus	ON989229
CY422	F	60-69	04/17	03/17	Cyprus	HC	10	20.00	Infected in Cyprus	ON989230
CY423	F	30-39	04/17	-/16	Cameroon	HC	578	32.30	Infected in Cameroon	ON989231
CY424	M	20-29	04/17	11/17	Cyprus	MSM	930	21.00	Infected in Cyprus	ON989232
CY425	M	40-49	04/17	03/17	Indonesia	MSM	314	14.70	Infected in Cyprus	ON989233
CY426	F	40-49	04/17	03/17	Cameroon	HBC	482	404.00	Infected in Cameroon	PP909550
CY427	M	30-39	04/17	03/17	Cyprus	HC	22	560.00	Infected in Greece	ON989234
CY439	M	40-49	05/17	05/17	Greece	HBC	459	9.11	Infected in Cyprus	ON989235
CY443	M	40-49	05/17	04/17	Bulgaria	MSM	27	149.00	Infected in Cyprus	ON989236

CY444	M	40-49	06/17	04/17	Greece	MSM	746	32.60	Infected in Cyprus	ON989237
CY447	M	30-39	06/17	01/17	Cyprus	MSM	571	13.20	Infected in Cyprus	ON989238
CY448	M	30-39	06/17	08/15	Cyprus	MSM	739	30.80	Infected in Cyprus	ON989239
CY449	M	40-49	06/17	05/17	UK	MSM	947	8.48	Infected in Cyprus	ON989240
CY450	M	20-29	06/17	05/17	Cyprus	MSM	525	138.00	Infected in Cyprus	ON989241
CY451	M	20-29	06/17	05/17	UK	HC/IDU	793	25.30	Infected in India	ON989242
CY452	M	30-39	06/17	02/13	Cyprus	MSM	1,130	22.30	Infected in Cyprus	ON989243
CY454	F	30-39	07/17	06/17	Romania	HC	459	65.80	Infected in Cyprus	PP909551
CY455	M	50-59	07/17	07/17	Cyprus	HC	51	96.70	Infected in Cyprus	ON989244
CY461	M	30-39	08/17	06/17	Cyprus	MSM	384	17.30	Infected in Cyprus	ON989247
CY462	M	40-49	08/17	07/17	Cyprus	MSM	344	2.00	Infected in Cyprus	ON989248
CY463	F	30-39	08/17	08/17	Bulgaria	HC	433	12.20	Infected in Bulgaria	ON989249
CY465	F	30-39	08/17	08/17	Romania	IDU	234	167.00	Infected in Cyprus	ON989250
CY466	M	40-49	09/17	08/17	Greece	MSM	189	17.70	Infected in Cyprus	ON989251
CY467	M	30-39	09/17	06/17	Nigeria	HBC	345	79.90	Infected in Nigeria	OK584018
CY470	M	50-59	09/17	07/17	Ivory Coast	HC	320	16.10	Infected in Ivory Coast	ON989252
CY471	M	40-49	09/17	06/17	Greece	MSM	509	12.30	Infected in Cyprus	ON989253
CY472	M	40-49	09/17	08/17	Romania	MSM	85	186.00	N/A	ON989254
CY473	M	40-49	09/17	06/17	Cyprus	MSM	502	112.00	Infected in Cyprus	ON989255
CY477	M	30-39	10/17	05/17	Hungary	HBC	538	269.00	Infected in UK	ON989256
CY478	M	50-59	10/17	08/17	Cyprus	MSM	205	145.00	Infected in Cyprus	ON989257
CY479	F	60-69	10/17	09/17	Sri Lanka	TR	463	29.30	Infected in Sri Lanka	ON989258
CY480	F	40-49	10/17	10/17	Cameroon	HC	237	33.40	Infected in Cyprus	ON989259
CY482	F	30-39	11/17	10/17	Cyprus	HC	4	537.00	Infected in Cyprus	ON989260
CY483	F	40-49	11/17	11/17	Cameroon	HC	394	27.30	Infected in Cameroon	ON989261
CY485	M	20-29	11/17	11/17	Cyprus	MSM	495	6.27	Infected in Cyprus	ON989262
CY486	M	30-39	11/17	09/17	Cyprus	MSM	470	17.50	Infected in Cyprus	ON989263
CY487	M	60-69	11/17	11/17	Cyprus	HC	662	31.80	Infected in Cyprus	ON989264
CY488	M	50-59	12/17	11/17	Cyprus	MSM/IDU	864	89.20	Infected in Cyprus	ON989265

CY489	M	50-59	12/17	11/17	UK	MSM	580	13.20	Infected in Cyprus	PP909552
CY490	F	40-49	12/17	12/17	Ukraine	HC/TR	43	640.00	Infected in Ukraine	ON989266
CY492	F	20-29	01/18	12/17	Romania	HC	487	8.98	Infected in Cyprus	ON989267
CY493	M	30-39	01/18	01/18	Romania	HC	1,446	47.70	N/A	ON989268
CY494	M	30-39	01/18	01/18	Cyprus	HC	310	217.00	Infected in Cyprus	OK283056
CY495	M	70-79	02/18	11/17	Cyprus	HBC	462	1.39	Infected in Cyprus	ON989269
CY496	M	40-49	02/18	11/17	Italy	HC	1,037	5.65	Infected in Cyprus	ON989270
CY497	F	40-49	02/18	11/17	Cameroon	HC	102	491.00	Infected in Cameroon	ON989271
CY499	M	40-49	02/18	12/17	Cyprus	HC	4	458.00	Infected in Cyprus	PP909553
CY504	M	20-29	03/18	01/18	Romania	MSM	568	73.50	Infected in Cyprus	ON989274
CY508	F	30-39	03/18	01/18	Nigeria	HC	198	53.00	Infected in Nigeria	ON989275
CY509	M	60-69	03/18	02/18	UK	MSM	538	39.10	Infected in Cyprus	ON989276
CY510	M	40-49	03/18	02/18	Cyprus	MSM	627	23.90	Infected in Cyprus	ON989277
CY512	M	60-69	03/18	03/18	Cyprus	MSM	150	0.32	Infected in Cyprus	OP781327
CY515	M	20-29	04/18	12/17	Romania	HBC	1,037	4.69	Infected in Spain	ON989278
CY516	F	40-49	04/18	03/18	Russia	HC	144	53.30	Infected in Cyprus	ON989279
CY517	M	60-69	04/18	04/18	Cyprus	MSM	26	402.00	Infected in Cyprus	PP909554
CY520	M	50-59	05/18	03/18	Cyprus	MSM	300	287.00	Infected in Cyprus	OK283057
CY523	M	30-39	05/18	04/18	Cyprus	MSM	579	3.23	Infected in Cyprus	ON989281
CY524	M	30-39	05/18	-/13	Cameroon	HC	327	121.00	Infected in Cameroon	PP909555
CY525	M	50-59	05/18	04/18	Cyprus	MSM	124	136.00	Infected in Greece	ON989282
CY526	M	20-29	05/18	04/18	Lebanon	MSM	631	6.96	Infected in Cyprus	ON989283
CY528	M	50-59	06/18	04/18	Cyprus	HC	726	50.40	Infected in Cyprus	ON989284
CY529	M	30-39	06/18	05/18	Greece	HBC	357	76.60	Infected in Greece	ON989285
CY530	F	40-49	06/18	05/18	Cameroon	HC	161	12.10	Infected in Cameroon	ON989286
CY533	M	30-39	07/18	05/18	Cyprus	HC	851	15.90	Infected in Cyprus	OK283058
CY534	M	20-29	07/18	05/18	Cyprus	MSM	498	13.90	Infected in Cyprus/Austria	ON989287
CY535	M	30-39	07/18	01/18	Cameroon	HC	776	28.10	Infected in Cameroon	ON989288
CY537	M	50-59	07/18	06/18	Cyprus	HC	729	146.00	Infected in Cyprus	ON989289

CY538	F	40-49	07/18	09/17	Cameroon	HC/TR	208	11.40	Infected in Cameroon	ON989290
CY539	M	30-39	07/18	11/17	Bulgaria	MSM	824	34.20	Infected in Cyprus	ON989291
CY541	F	60-69	08/18	-/88	Cyprus	HC	92	57.70	Infected in Greece	ON989292
CY542	M	20-29	08/18	06/18	Greece	MSM	482	22.10	Infected in Greece	ON989293
CY543	M	30-39	08/18	03/18	Cyprus	MSM	1,079	30.30	Infected in Cyprus	ON989294
CY544	M	30-39	08/18	07/18	Cyprus	MSM	1,215	21.30	Infected in Cyprus	ON989295
CY545	M	30-39	08/18	-/18	Cyprus	MSM	932	5.81	Infected in Cyprus	ON989296
CY546	M	40-49	08/18	07/18	Cyprus	MSM	256	4.10	Infected in Cyprus	ON989297
CY551	M	30-39	09/18	08/18	Greece	MSM	594	6.85	Infected in Cyprus	ON989299
CY552	M	30-39	09/18	08/18	Cyprus	HBC	457	437.00	Infected in Cyprus	ON989300
CY553	M	40-49	10/18	09/16	Romania	HC	36	1,700.00	Infected in Cyprus	ON989301
CY554	M	30-39	10/18	06/18	UK	MSM	1,034	377.00	Infected in Cyprus	ON989302
CY555	M	30-39	10/18	09/18	Philippines	HC	502	22.80	Infected in Cyprus	ON989303
CY556	M	30-39	10/18	02/18	Cyprus	MSM	470	1,090.00	N/A	ON989304
CY557	M	30-39	10/18	09/18	Romania	HC	208	1,020.00	Infected in Cyprus	ON989305
CY559	M	50-59	11/18	09/18	Cyprus	MSM	420	64.20	Infected in Cyprus	ON989306
CY562	M	30-39	11/18	10/18	Cyprus	MSM	939	454.00	Infected in Cyprus	ON989308
CY563	M	40-49	11/18	10/18	Cyprus	MSM	1,006	54.20	Infected in Cyprus	ON989309
CY564	M	20-29	11/18	02/18	Cyprus	MSM	812	1.46	Infected in Cyprus	ON989310
CY565	M	30-39	11/18	05/18	Romania	HC	9	453.00	Infected in Romania	ON989311
CY571	M	30-39	11/18	09/18	Cameroon	HC/OHPC	525	45.00	Infected in Cameroon	ON989312
CY572	M	20-29	12/18	10/18	Cyprus	MSM	741	16.30	Infected in Cyprus	ON989313
CY574	M	30-39	12/18	10/18	Cameroon	HC	315	30.70	Infected in Cameroon	PP909556
CY575	M	50-59	12/18	11/18	Cyprus	MSM	718	6.21	Infected in Cyprus	ON989314
CY576	F	20-29	12/18	10/18	Cameroon	HC	42	656.00	Infected in Cameroon	ON989315
CY579	M	40-49	01/19	-/18	Cameroon	HC/TR	879	83.30	Infected in Cameroon	ON989316
CY583	M	30-39	01/19	12/19	Cyprus	MSM	278	742.00	Infected in Cyprus	ON989319
CY584	M	50-59	01/19	01/19	Cyprus	MSM	9	3.76	Infected in Venezuela	ON989320
CY585	F	40-49	01/19	12/18	Cameroon	HC	294	44.40	Infected in Cameroon	ON989321

CY587	M	30-39	01/19	12/18	Cyprus	MSM	417	77.90	Infected in Cyprus	ON989322
CY591	M	30-39	02/19	12/18	Cyprus	MSM	845	44.30	Infected in Cyprus	ON989323
CY593	M	30-39	02/19	-/19	Cyprus	MSM	34	402.00	N/A	ON989324
CY594	M	30-39	02/19	01/19	Cyprus	HC	772	16.20	Infected in Cyprus	ON989325
CY599	M	40-49	03/19	-/19	Nigeria	HC	334	19.80	Infected in Cameroon	ON989326
CY600	M	30-39	03/19	01/19	Cyprus	MSM	318	<0.02	Infected in Cyprus	PP909557
CY601	M	70-79	04/19	-/19	UK	HC	111	1,380.00	Infected in Zimbabwe	ON989327
CY602	M	40-49	04/19	01/19	Cameroon	HC	540	31.00	Infected in Cameroon	ON989328
CY605	M	30-39	04/19	03/19	Cyprus	MSM	1,185	46.60	N/A	ON989330
CY606	M	20-29	04/19	02/19	Cameroon	HC	134	105.00	Infected in Cameroon	PP909558
CY607	M	50-59	05/19	04/19	Cyprus	HBC	79	68.20	N/A	ON989331
CY609	M	30-39	05/19	04/19	Cyprus	MSM	675	29.80	Infected in UK	ON989332
CY611	F	40-49	05/19	03/19	Cameroon	HC	7	12.50	Infected in Cameroon	ON989333
CY612	M	30-39	05/19	03/19	Cyprus	MSM	965	26.50	Infected in UK	ON989334
CY614	M	20-29	06/19	04/19	Cyprus	MSM	237	1,260.00	Infected in Cyprus	OK283059
CY615	M	30-39	06/19	06/19	Greece	IDU	996	1,860.00	Infected in Cyprus	ON989335
CY616	M	40-49	06/19	06/19	Cyprus	MSM	145	135.00	Infected in Cyprus	ON989336
CY617	M	40-49	07/19	04/19	Cyprus	HBC	1,304	17.30	Infected in Cyprus	ON989337
CY619	M	30-39	07/19	07/19	Cameroon	HC	393	99.40	Infected in Cameroon	ON989338
CY620	M	40-49	07/19	05/19	Cyprus	HC	450	124.00	Infected in Cyprus	ON989339
CY621	M	40-49	07/19	06/19	Cyprus	MSM	469	203.00	Infected in Cyprus	PP909559
CY622	M	70-79	07/19	05/19	UK	MSM	694	87.80	N/A	OK283060
CY624	F	20-29	08/19	05/19	Cameroon	HC	575	529.00	Infected in Cameroon	ON989340
CY625	M	30-39	08/19	07/19	Cyprus	HBC	406	269.00	Infected in Cyprus	ON989341
CY630	M	70-79	09/19	-/19	Cyprus	MSM	294	324.00	Infected in Cyprus	OK283061
CY631	M	30-39	09/19	07/19	Cyprus	MSM	316	237.00	Infected in Cyprus	PP909560
CY632	F	20-29	09/19	08/19	Cameroon	HC	555	250.00	Infected in Cameroon	PP909561
CY633	F	30-39	09/19	06/19	Cameroon	HC	586	4.92	Infected in Cameroon	PP909562
CY635	F	30-39	10/19	07/19	Cameroon	HC	408	24.60	Infected in Cameroon	PP909563
CY636	F	30-39	10/19	04/19	Russia	HC	59	872.00	Infected in Russia	PP909564

CY637	F	30-39	10/19	07/19	Cameroon	HBC	186	452.00	Infected in Cameroon	PP909565
CY638	M	30-39	10/19	08/19	Nigeria	HC	445	11.70	Infected in Nigeria	PP909566
CY639	M	30-39	10/19	08/19	Cameroon	MSM/IDU	388	219.00	Infected in Cyprus	OP894080
CY640	M	40-49	10/19	09/19	Cyprus	MSM	426	317.00	Infected in Cyprus	OK283062
CY641	M	40-49	11/19	09/19	Greece	MSM	346	34.70	Infected in Cyprus	PP909567
CY642	M	20-29	11/19	-/19	Cyprus	MSM	3	792.00	Infected in Cyprus/UK	PP909568
CY643	F	30-39	11/19	09/19	Cameroon	HC	246	180.00	Infected in Cameroon	PP909569
CY646	F	40-49	11/19	10/19	Cameroon	HC	N/A	59.90	Infected in Cameroon	PP909570
CY650	M	20-29	12/19	-/19	Cameroon	HC	2,583	1.91	Infected in Cameroon	PP909571
CY652	M	20-29	12/19	12/19	Cyprus	MSM	969	8.54	Infected in Cyprus	PP909572
CY655	F	30-39	01/20	12/19	Cameroon	HC	184	42.50	Infected in Cameroon	PP909573
CY656	M	30-39	01/20	10/19	Cameroon	HC	464	48.60	Infected in Cameroon	PP909574
CY657	M	30-39	01/20	10/19	Romania	MS	634	0.82	Infected in Cyprus	PP909575
CY658	M	30-39	01/20	11/19	Greece	MSM	459	6.92	Infected in Cyprus	PP909576
CY659	F	20-29	01/20	11/19	Romania	HC/ IDU	744	12.80	Infected in Cyprus	PP909577
CY660	F	30-39	01/20	12/19	Rwanda	HC	264	380.00	Infected in Rwanda	PP909578
CY662	F	40-49	02/20	12/19	Cameroon	HC	310	41.80	Infected in Cameroon	PP909579
CY664	F	30-39	02/20	09/19	Cameroon	HC	931	1.69	Infected in Cameroon	PP909580
CY665	M	30-39	02/20	-/19	Cameroon	HC	390	34.60	Infected in Cameroon	PP909581
CY666	M	20-29	02/20	01/20	Rwanda	MSM	477	20.10	Infected in Turkey	PP909582
CY667	F	30-39	02/20	12/19	Cameroon	HC	423	126.00	Infected in Cameroon	PP909583
CY668	F	80-89	02/20	01/20	Cyprus	HC	275	130.00	Infected in Cyprus	PP909584
CY669	M	70-79	02/20	02/20	Cyprus	HBC	303	144.00	Infected in Cyprus	PP909585
CY670	M	50-59	02/20	01/20	Bulgaria	MSM	103	463.00	Infected in Cyprus	OK283063
CY671	M	30-39	02/20	12/19	Cyprus	MSM	534	145.00	Infected in Cyprus/Holland	PP909586
CY672	M	20-29	02/20	10/29	Cameroon	MSM	236	387.00	Infected in Cameroon	PP909587
CY673	M	30-39	03/20	-/19	Zimbabwe	HC	322	31.50	Infected in Zimbabwe	PP909588
CY674	M	40-49	03/20	02/20	Cyprus	IDU	318	N/A	Infected in Cyprus	PP909589
CY675	M	20-29	03/20	01/20	Congo	HC	273	18.30	Infected in South Africa	PP909590

CY676	M	20-29	03/20	11/19	Colombia	HBC	773	27.70	Infected in Cyprus	PP909591
CY677	M	50-59	03/20	-/20	UK	MSM/IDU	246	25.10	Infected in Spain/Portugal/Bulgaria	PP909592
CY678	M	50-59	03/20	03/20	Cyprus	MSM	1,608	22.90	Infected in Cyprus	PP909593
CY679	F	20-29	04/20	03/20	Sierra Leone	HC	N/A	92.50	Infected in Sierra Leone	PP909594
CY680	M	20-29	04/20	12/19	Turkey	HC	313	85.80	Infected in Turkey	PP909595
CY681	M	70-79	04/20	-/20	UK	MSM	946	12.80	Infected in Cyprus	PP909596
CY682	F	40-49	04/20	03/20	Cameroon	HC	457	97.50	Infected in Cameroon	PP909597
CY683	M	30-39	04/20	-/20	Cyprus	HC	603	259.00	Infected in Cyprus	PP909598
CY684	M	50-59	04/20	02/20	Greece	MSM	657	13.40	Infected in Cyprus	PP909599
CY685	F	20-29	04/20	01/20	Cameroon	HC	884	31.50	Infected in Cameroon	PP909600
CY686	M	60-69	05/20	05/20	UK	MSM	131	1,070.00	Infected in Cyprus	OK283064
CY687	F	30-39	05/20	03/18	Cameroon	HC	490	21.70	Infected in Cameroon	PP909601
CY691	M	30-39	05/20	03/20	Cyprus	MSM	536	41.10	Infected in Cyprus	PP909602
CY692	M	40-49	06/20	05/20	Cyprus	MSM	577	32.40	Infected in Cyprus	PP909603
CY693	M	20-29	06/20	01/20	Sierra Leone	HC	794	32.50	Infected in Sierra Leone	PP909604
CY694	M	20-29	06/20	05/20	Cyprus	MSM	20	639.00	Infected in Cyprus	PP909605
CY695	F	50-59	06/20	06/20	Georgia	HC	9	37.70	Infected in Georgia	PP909606
CY696	M	30-39	06/20	06/20	Cyprus	N/A	14	167.00	N/A	PP909607
CY697	M	30-39	06/20	04/20	Cyprus	MSM	500	73.10	Infected in Cyprus	OP781329
CY699	F	20-29	06/20	06/20	Ukraine	HC	585	22.90	Infected in Ukraine	PP909608
CY700	M	40-49	06/20	06/20	Georgia	N/A	N/A	N/A	N/A	PP909609
CY701	M	20-29	06/20	06/20	Greece	MSM	699	30.20	Infected in Greece	PP909610
CY703	M	30-39	07/20	03/20	Cyprus	MSM	645	33.60	Infected in Cyprus	PP909611
CY704	M	40-49	07/20	06/20	Cyprus	MSM	137	23.90	Infected in Cyprus	PP909612
CY705	M	50-59	07/20	06/20	Bulgaria	MSM	235	41.40	Infected in Cyprus	PP909613
CY707	M	20-29	07/20	05/20	Pakistan	MSM	581	50.00	Infected in Cyprus	PP909614
CY708	F	30-39	07/20	05/20	Cameroon	HC	147	12.60	Infected in Cameroon	PP909615
CY709	M	40-49	07/20	06/20	Cyprus	MSM	441	12.00	Infected in Cyprus	PP909616
CY710	M	40-49	07/20	06/20	Romania	HBC	330	55.10	Infected in Romania	OP781330

CY711	M	30-39	07/20	06/20	Cyprus	MSM	25	50.60	Infected in Cyprus	PP909617
CY712	M	20-29	07/20	06/20	Cyprus	MSM	665	129.00	Infected in Cyprus	PP909618
CY716	F	50-59	07/20	-/20	Bulgaria	HC	24	153.00	Infected in Bulgaria	PP909619
CY717	F	40-49	07/20	06/20	Georgia	HC	159	13.00	Infected in Georgia	PP909620
CY718	M	40-49	07/20	03/20	Cyprus	HC	346	10.60	Infected in Cyprus	PP909621
CY719	M	30-39	07/20	12/19	Cyprus	MSM	669	311.00	Infected in Cyprus	PP909622
CY720	M	50-59	08/20	07/20	Cyprus	HC	561	44.90	Infected in South Africa	PP909623
CY721	F	30-39	08/20	06/20	Somalia	HC/TR	159	1,920.00	Infected in Somalia	PP909624
CY722	F	40-49	08/20	07/20	Cameroon	HC	207	2.08	Infected in Cameroon	PP909625
CY723	M	30-39	08/20	-/20	Cyprus	MSM	395	232.00	Infected in Cyprus	PP909626
CY724	M	40-49	09/20	03/20	Cyprus	MSM	497	287.00	Infected in Cyprus	PP909627
CY725	M	40-49	09/20	07/20	Cyprus	HBC	169	438.00	Infected in Cyprus	PP909628
CY726	M	30-39	09/20	-/18	Cyprus	MSM	887	53.50	Infected in Germany	PP909629
CY727	M	30-39	09/20	-/20	Cyprus	MSM	364	17.00	Infected in Cyprus	PP909630
CY728	M	40-49	09/20	07/20	Poland	HBC	339	19.60	Infected in Cyprus	PP909631
CY730	F	30-39	09/20	03/20	Cameroon	HC	534	100.00	Infected in Cameroon	PP909632
CY731	M	20-29	09/20	-/20	Cyprus	HC	476	24.00	Infected in Cyprus	PP909633
CY732	F	40-49	09/20	08/20	Moldova	HC	543	18.10	Infected in Cyprus	PP909634
CY733	M	40-49	09/20	09/20	Cyprus	MSM	30	153.00	Infected in Cyprus	PP909635
CY734	M	20-29	09/20	-/20	Cyprus	HC	562	30.30	Infected in Greece	PP909636
CY736	M	30-39	10/20	-/20	Cyprus	MSM	589	49.40	Infected in Cyprus	PP909637
CY737	F	40-49	10/20	08/20	Cameroon	HC	586	33.40	Infected in Cameroon	PP909638
CY738	F	40-49	10/20	08/20	Ukraine	HC	202	9,910.00	N/A	PP909639
CY740	M	30-39	10/20	07/20	Cameroon	HC	207	13.70	Infected in Cameroon	PP909640
CY741	F	40-49	10/20	09/20	Nigeria	HC	128	89.10	N/A	PP909641
CY742	M	40-49	11/20	09/20	Cyprus	HBC	309	864.00	Infected in Cyprus	OK283065
CY743	M	50-59	11/20	10/20	Cyprus	MSM	113	374.00	Infected in Greece	PP909642
CY744	F	20-29	11/20	10/20	Cameroon	HC	674	28.00	Infected in Cameroon	PP909643
CY746	M	50-59	11/20	04/20	UK	MSM	654	252.00	Infected in Philippines	PP909644

CY750	M	40-49	12/20	11/20	Cyprus	MSM	429	126.00	Infected in Cyprus	PP909645
CY751	M	30-39	12/20	11/20	Russia	MSM	436	40.10	Infected in Russia	PP909646
CY754	M	50-59	12/20	11/20	Cyprus	MSM	652	126.00	Infected in Cyprus	PP909647
CY755	M	30-39	12/20	11/20	Cyprus	MSM	532	76.40	Infected in Cyprus	PP909648
CY756	M	20-29	12/20	12/20	Congo	MSM	155	0.47	N/A	PP909649
CY757	F	30-39	01/21	01/21	Guinea	HC	205	108.00	Infected in Guinea	PP909650
CY760	M	40-49	01/21	-/21	Slovakia	MSM	746	132.00	Infected in Cyprus	PP909651
CY762	M	60-69	01/21	01/21	Cyprus	HC	111	134.00	Infected in Cyprus	PP909652
CY763	M	30-39	02/21	01/21	Cyprus	MSM	404	94.00	Infected in Cyprus	PP909653
CY764	M	20-29	02/21	01/21	Romania	MSM	289	63.10	Infected in Malta	PP909654
CY765	M	40-49	02/21	-/21	Romania	MSM	698	24.00	Infected in Malta	PP909655
CY766	M	50-59	02/21	02/21	Cyprus	HBC	166	31.00	Infected in Cyprus	PP909656
CY768	F	40-49	02/21	-/19	Cameroon	HC	665	1.73	Infected in Cameroon	PP909657
CY769	F	40-49	02/21	-/05	Cameroon	HC	574	<0.02	Infected in Cameroon	PP909658
CY770	M	20-29	02/21	02/21	Cyprus	MSM	1,147	30.00	Infected in Cyprus	PP909659
CY772	F	40-49	02/21	-/21	Cameroon	HC	206	52.40	Infected in Cameroon	PP909660
CY773	M	20-29	02/21	-/19	Cameroon	HC	189	52.40	Infected in Cameroon	PP909661
CY774	M	30-39	02/21	02/21	Sierra Leone	N/A	498	12.90	Infected in Sierra Leone	PP909662
CY775	M	50-59	03/21	12/21	Cyprus	MSM	443	24.40	Infected in Greece/Cyprus	PP909663
CY776	F	20-29	03/21	03/21	Congo	HC	448	15.30	Infected in Congo	PP909664
CY777	M	40-49	03/21	12/20	Cyprus	MSM	605	43.40	Infected in Greece	PP909665
CY778	M	40-49	03/21	10/10	Cyprus	MSM	35	326.00	Infected in UK	PP909666
CY779	M	60-69	03/21	-/21	Cyprus	MSM	990	15.50	Infected in Cyprus	PP909666
CY780	M	40-49	04/21	03/21	Poland	MSM	314	231.00	Infected in Cyprus	PP909668
CY781	M	20-29	04/21	02/21	Cyprus	MSM	485	84.70	Infected in Germany/Greece	PP909669
CY782	M	60-69	04/21	03/21	Cyprus	MSM	208	24.30	Infected in Cyprus	PP909670
CY785	M	20-29	04/21	03/21	Cameroon	HC	367	27.90	Infected in Cameroon	PP909671
CY786	M	30-39	04/21	03/21	Cameroon	MSM	478	55.70	Infected in Cameroon	PP909672
CY787	M	20-29	04/21	03/21	Nigeria	MSM	494	5.25	Infected in Cyprus	PP909673
CY788	M	20-29	04/21	03/21	Nigeria	MSM	704	26.00	Infected in Cyprus	PP909674

CY790	M	40-49	04/21	04/21	Greece	MSM	173	3,100.00	Infected in Cyprus	PP909675
CY792	M	30-39	05/21	04/21	Bulgaria	MSM	742	4.62	Infected in Cyprus	PP909676
CY793	F	30-39	05/21	04/21	Sierra Leone	HC	802	<0.02	Infected in Cyprus	PP909677
CY794	M	40-49	05/21	04/21	Cyprus	MSM	139	448.00	Infected in Israel	PP909678
CY795	M	20-29	05/21	03/21	Sierra Leone	HC	344	24.30	Infected in Sierra Leone	PP909679
CY796	M	40-49	05/21	04/21	Cyprus	MSM	200	17.90	Infected in Cyprus	PP909680
CY797	M	30-39	05/21	04/21	Bulgaria	MSM	679	0.73	Infected in Cyprus	PP909681
CY798	M	20-29	05/21	04/21	Sierra Leone	MSM	400	629.00	Infected in Sierra Leone	PP909682
CY799	M	40-49	05/21	04/20	UK	MSM	888	4.19	Infected in Cyprus	PP909683
CY800	M	30-39	05/21	04/20	Poland	MSM	442	67.50	Infected in Cyprus	PP909684
CY801	M	30-39	05/21	04/20	Cyprus	MSM	82	279.00	Infected in Cyprus	PP909685
CY802	F	30-39	05/21	05/21	Cyprus	HC	874	66.80	Infected in Cyprus	PP909686
CY803	M	40-49	05/21	05/21	Cyprus	MSM	995	26.50	Infected in Cyprus	PP909687
CY805	M	30-39	06/21	05/21	Cyprus	MSM	466	14.60	Infected in Cyprus	OP894081
CY806	M	70-79	06/21	05/21	Cyprus	MSM	133	827.00	Infected in Cyprus	PP909688
CY807	M	40-49	06/21	06/21	Ireland	MSM	2	N/A	Infected in Cyprus	PP909689
CY808	M	40-49	06/21	05/21	Cyprus	HC	81	218.00	Infected in Cyprus	PP909690
CY809	F	30-39	06/21	05/21	Cyprus	HC	381	43.90	Infected in Cyprus	PP909691
CY810	F	20-29	06/21	05/21	Sierra Leone	HC	897	1.62	Infected in Sierra Leone	PP909692
CY811	F	30-39	06/21	05/21	UK	HC	30	N/A	Infected in Cyprus	PP909693
CY812	M	20-29	06/21	06/21	Cameroon	MSM	513	39.40	Infected in Cyprus	PP909694
CY813	M	40-49	06/21	06/21	Cyprus	HC	294	76.26	Infected in Cyprus	PP909695
CY814	F	30-39	06/21	06/21	Ukraine	HC	10	658.00	Infected in Ukraine	PP909696
CY815	M	30-39	06/21	06/21	Cyprus	MSM	654	10.00	Infected in Cyprus	PP909697
CY816	M	30-39	06/21	-/19	Cameroon	HC	335	17.00	Infected in Cameroon	PP909698
CY817	M	30-39	06/21	-/21	UK	MSM	599	52.70	Infected in Cyprus	PP909699
CY818	M	30-39	06/21	05/21	Cyprus	MSM	739	80.10	Infected in Cyprus	PP909700
CY820	M	60-69	07/21	06/21	Cyprus	HBC	794	41.80	Infected in Cyprus	PP909701
CY821	M	40-49	07/21	06/21	Cyprus	MSM	1,361	190.00	Infected in Cyprus	PP909702
CY822	M	40-49	07/21	06/21	Cyprus	MSM	541	77.60	Infected in Cyprus	PP909703

CY824	M	50-59	07/21	07/21	Cyprus	MSM	13	9,000.00	Infected in Cyprus	OP894082
CY825	M	40-49	07/21	08/20	Romania	HC/IDU	23	690.00	Infected in Romania	PP909704
CY826	M	50-59	07/21	07/21	Bulgaria	MSM	132	5,310.00	Infected in Cyprus	PP909705
CY828	M	30-39	08/21	06/21	Nigeria	HC	540	11.70	N/A	PP909706
CY829	M	30-39	08/21	07/21	Sierra Leone	HC	136	1,110.00	Infected in Sierra Leone	PP909707
CY830	M	60-69	08/21	08/21	Cyprus	HC	17	4,120.00	Infected in Cyprus	PP909708
CY831	M	40-49	08/21	07/21	Cyprus	MSM	544	3.72	Infected in Cyprus	PP909709
CY836	M	50-59	08/21	07/21	Cyprus	MSM	97	523.00	Infected in Cyprus	PP909710
CY838	M	40-49	09/21	09/21	Nigeria	HC	391	521.00	Infected in Nigeria	PP909711
CY839	M	20-29	09/21	08/21	Somalia	HC	595	0.11	Infected in Somalia	PP909712
CY840	M	20-29	09/21	08/21	Congo	HC	453	185.00	Infected in Congo	PP909713
CY841	M	20-29	09/21	04/21	Nigeria	MSM	429	19.23	Infected in Nigeria	PP909714
CY842	M	40-49	09/21	09/21	Cyprus	HC	735	48.70	Infected in Cyprus	OP894083
CY843	M	60-69	09/21	09/21	Cyprus	HC	774	515.14	Infected in Cyprus	PP909715
CY844	M	40-49	10/21	10/21	Cyprus	MSM	208	136.39	N/A	PP909716

[a] Indicates the laboratory code for each study participant. [b] M, male; F, female [c] Indicates the date (month/year; -, unknown month) of the first known positive HIV antibody test. [d] Country of birth of the study participants; UK, United Kingdom. [e] HBC, homo/bisexual contact; HC, heterosexual contact; MSM, men who have sex with men; IDU, injecting drug user; TR, blood transfusion; OHPC, originating from high prevalence country [f] Information provided by the study participants. N/A, Not available.
